# Nanoparticle-Catalysed Microwave-Driven MCRs for Sustainable Heterocycle Synthesis

**DOI:** 10.3390/molecules31061031

**Published:** 2026-03-19

**Authors:** Venkatesan Kasi, Malgorzata Jeleń, Xiao-Hui Chu, Parasuraman Karthikeyan, Beata Morak Młodawska, Lai-Hock Tey

**Affiliations:** 1 Chemistry Division, Department of H&S, CVR College of Engineering, Vastunagar, Ibrhimpatnam 501510, Telangana, India; 2 Department of Organic Chemistry, Faculty of Pharmaceutical Sciences, The Medical University of Silesia, Jagiellońska 4, 41-200 Sosnowiec, Polandbmlodawska@sum.edu.pl (B.M.M.); 3 Department of Chemical Science, Faculty of Science, Universiti Tunku Abdul Rahman (UTAR), Kampar Campus, Jalan Universiti, Kampar 31900, Malaysia; 4 PG and Research Department of Chemistry, Pachaiyappas College Campus, University of Madras, Chennai 600030, Tamil Nadu, India

**Keywords:** nanoparticle catalysis, microwave MCRs, green synthesis, sustainable heterocycles, recyclable catalysts, green chemistry

## Abstract

Nanoparticle-catalysed microwave-aided multicomponent reactions (MCRs) have been demonstrated to be competent and environmentally benign tools for the quick synthesis of a wide spectrum of fused heterocyclic systems. The distinctive physicochemical properties of nanoparticles, including a substantial surface area, readily modifiable surface functionality, and heightened catalytic activities, when coupled with microwave irradiation, have enabled a marked improvement in reaction rates, product yields, and selectivity compared to conventional heating methods. This review highlights recent advancements in microwave-assisted MCRs facilitated by diverse nanomaterials, such as magnetic nanocatalysts, metal and metal oxide nanoparticles, mesoporous silica systems, and nanohybrids. It emphasises catalyst design, catalytic efficacy, scope, recyclability, and alignment with green chemistry principles in both solvent-free and aqueous environments, as well as the utilisation of recyclable catalysts. In summary, microwave-assisted multi-component reactions catalysed by nanoparticles are ecofriendly and versatile methods for the sustainable synthesis of such fused heterocycles containing bioactive pyridine, pyrazole, phenazine, pyrimidine, pyran, imidazole, and relevant pyridine derivatives, possessing potential in medicinal and material chemistry.

## 1. Introduction

MCRs are among the most powerful and efficient methods available in modern organic chemistry for the one-step assembly of complex molecules from at least three different starting materials [1,2,3]. MCRs have developed into a powerful toolkit for rapid molecular assembly, which is highly atom-economical and produces less waste, thus providing an efficient method for sustainable and “green chemistry” applications compared to traditional multi-step syntheses [4,5,6]. According to the literature, MCRs have attracted significant research attention for the preparation of pharmacologically active heterocyclic derivatives and highly functionalized organic molecules, owing to their environmentally benign nature, which minimizes reaction steps, energy requirements, and waste generation [7,8,9]. Over the past few decades, MCRs have found many applications in the preparation of heterocyclics, which are found in the core structure of many medicinal compounds, agrochemicals, and functional materials [10,11,12]. Their tolerance capacity, diversity, and compatibility with environmentally friendly conditions have made them especially useful in the field of medicinal and materials chemistry [13,14].

Although several reviews have discussed microwave-assisted MCRs or nanoparticle catalysis independently, a systematic integration of nanoparticle-catalysed microwave-driven MCRs focusing on sustainability metrics, catalyst recyclability, and heterocycle diversity remains underexplored.

### 1.1. Role of Nanoparticle Catalysts in Sustainable MCRs

Catalysis is one of the important aspects of MCRs, and researchers have been focusing on increasing their efficiency and sustainability with the help of catalysis. In this regard, nanoparticle catalysts have garnered considerable interest due to their specific physicochemical attributes, such as high surface area and high approachability of active sites, in contrast with both homogeneous and heterogeneous catalysts. They can easily overcome the limitations of homogeneous catalysts and the difficulties of separation associated with heterogeneous catalysts.

Metal, metal oxide, silica-supported, and magnetic nanoparticles have been extensively investigated in recent years as effective catalysts for MCRs, leading to higher yields, faster reaction times, and higher selectivity [15,16,17,18]. Moreover, magnetic nanocatalysts exhibit peculiar advantages such as facile separation in an external magnetic field and high recyclability, rendering them appealing from both industrial and ecological standpoints [19,20,21,22].

Furthermore, heterojunction interfaces in bimetallic or ternary systems enhance microwave absorption efficiency and localized heating effects, which can accelerate reaction kinetics and reduce activation energy barriers. Ternary metal oxides and alloy nanocomposites also demonstrate improved structural robustness and resistance to metal leaching, leading to better recyclability under repeated microwave cycles [23,24].

### 1.2. Microwave Irradiation as a Facilitating Eco-Friendly Method

Recently, MAOS has emerged as a revolutionary approach for the acceleration of chemical reactions by extending fast and uniform volumetric heating. At specific frequencies, the conversion of electromagnetic energy to thermal energy via a non-contact energy transfer procedure in MW irradiation supports high heating rates. In comparison to conventional thermal methods, microwave irradiation enables direct interaction with polar molecules and catalysts, which improves reaction kinetics coupled with better energy efficiency [25,26,27].

The microwave-supported MCRs of heterocycles have illustrated the substantial influence of microwave radiation in the quick, efficient, and sustainable creation of various heterocyclic structures. These advances highlight the potential of microwave-assisted approaches for complex molecular architecture and a broad range of pharmaceutical and medicinal chemistry research applications [28,29,30,31]. The application of microwave irradiation to MCRs has led to some striking improvements, including drastic reductions in reaction time, higher product yields, and limited use of solvents. Thus, microwave-aided MCRs form a significant area of green synthesis, particularly when combined with recyclable and heterogeneous catalytic systems.

### 1.3. Nanoparticle-Catalysed Microwave-Assisted MCRs for Heterocycle Synthesis

The combination of nanoparticle catalysis and microwave irradiation has recently revealed new opportunities for the sustainable preparation of heterocycles through MCRs. Indeed, this combination allows one to benefit from the advantages offered by both processes, which may enhance catalytic activities, mass transfer, and heating transfer compared to the conventional heating process [32,33].

Silica nanoparticles, metal oxide nanocatalysts, magnetic nanoparticles, as well as nanocomposites have been recently employed for the preparation of a diversity of heterocyclic frameworks, such as pyridines, pyrimidinones, xanthenes, quinazolines, azlactones, and fused nitrogen-containing heterocycles, using microwave irradiations. Their procedures often comply with the strategies of green chemistry in terms of recyclability and reaction medium, as reported in recent publications [34,35,36]. The emergence of nanoparticle-catalysed MCR reactions utilizing microwaves satisfies all the principles of green chemistry, which involve preventing the formation of waste, minimizing energy, and enabling catalyst reuse, especially in the context of preventing the wastage of materials or energy [37,38,39,40,41]. Hence, it could be concluded that nanoparticle-catalysed microwave-assisted MCRs are promising and environmentally benign methods for the synthesis of bioactive heterocycles.

### 1.4. Purpose and Scope of the Current Review

This review critically summarizes recent developments in nanoparticle-catalysed microwave-assisted MCRs for the green synthesis of heterocyclic compounds, based on literature published between 2017 and 2026. Recent developments also point out the usage of nanoparticles as catalysts in multicomponent reactions, focusing on both their high catalytic activity and sustainability in environmental terms.

## 2. Synthesis of Nanoparticles: Approaches and Sustainable Methods

The synthesis of nanoparticles has attracted considerable academic interest due to the unique physicochemical properties of nanomaterials, such as morphology, high surface area, and catalytic activity. Conventional synthesis routes, such as sol-gel, co-precipitation, chemical reduction, hydrothermal, and microemulsion methods, allow for the controlled synthesis of nanoparticles in terms of size, shape, and composition. These routes have been extensively used for the synthesis of metal oxides, metals, magnetic materials, and hybrid nanoparticles for catalytic, energy, environmental, and biomedical applications. However, most conventional routes are associated with the use of toxic solvents, hazardous reducing agents, and energy-intensive conditions.

As a result of these drawbacks, green and sustainable methods of nanoparticle synthesis have been developed as effective alternatives [42,43,44]. These approaches use eco-friendly solvents, green resources, and non-toxic reducing or stabilizing agents such as plant extracts, biopolymers, enzymes, and microorganisms [45,46]. Phytochemical-assisted synthesis is one such method that provides a simple and inexpensive approach, where natural compounds act as both reducing and capping agents to produce stable and biocompatible nanoparticles [47,48,49]. As a result, sustainable nanoparticle synthesis has been at the forefront of next-generation nanomaterials research. Recent studies have demonstrated that agricultural waste-derived phytochemicals, such as papaya leaf extracts and durian husk extracts, can effectively mediate the green synthesis of copper oxide nanoparticles with controlled morphology, high crystallinity, and excellent catalytic and environmental remediation performance, highlighting their sustainability and scalability [50].

## 3. Understanding the Catalytic Function of Nanoparticles

Nanoparticles occupy a central position in catalytic chemistry because their small sizes provide an extremely large surface area. Due to the availability of such a high number of surface atoms, the adsorption and activation of reactants take place easily. The small-sized particles contain their surface atoms in an unsaturated state, and their unique surface characteristics, such as edges and corners, show better efficacy in facilitating bond polarization, electron transfer, and intermediate stabilization compared to bulk particles. In the case of metal-oxide and metal nanoparticles, the catalytic phenomenon can depend on surface redox reactions, Lewis acid and base interactions, and the creation of reactive sites that reduce activation energy barriers and increase the rate of reactions.

In addition, nanoparticles are ideal platforms that allow synergistic processes involving the catalytic phase and support materials. Consider magnetic nanoparticles, which combine catalytic activity and easy recovery. Functionalized or doped nanoparticles may be used to control electronic properties and enhance selectivity. Green-synthesised metal oxide nanoparticles derived from plant extracts exhibit abundant surface defects and functional phytochemical capping layers, which enhance adsorption–desorption kinetics and facilitate electron transfer processes during catalytic transformations [51,52,53].

On the other hand, in heterogeneous catalysis, the catalytic functions of magnetic nanoparticles rely on adsorption and desorption cycling, intermediate formation at the surface, and selective product desorption, thus enhancing catalytic efficiency and ease of recovery. Furthermore, their non-aggregation and stabilized surface further indicate the importance of nanoparticles in the design of efficient and recoverable catalytic systems.

## 4. Microwave–Nanoparticle Interaction at the Molecular Level

Microwave irradiation can interact strongly with nanoparticle catalysts due to their unique dielectric and conductive properties. At the molecular level, nanoparticles can absorb microwave energy through dipolar polarization and ionic conduction mechanisms, which results in the rapid conversion of electromagnetic energy into thermal energy. Unlike conventional heating, where heat is transferred from the vessel surface to the reaction mixture, microwave irradiation produces volumetric and localized heating, often generating micro-scale “hot spots” on nanoparticle surfaces. These localized thermal gradients can significantly accelerate catalytic reactions by enhancing the adsorption of reactants and lowering activation barriers. In addition, the strong electromagnetic field may influence electron density distribution on nanoparticle surfaces, facilitating surface redox processes, polarization of functional groups, and stabilization of reaction intermediates. Consequently, the synergistic combination of microwave irradiation and nanoparticle catalysts promotes faster reaction kinetics, improved selectivity, and enhanced catalytic efficiency in multicomponent reactions.

The advantages of nanoparticle catalysts and a comparison between conventional catalysts and nanoparticle-based catalytic systems are summarized in Table 1. Due to their high surface area and enhanced catalytic activity, nanoparticle catalysts often exhibit improved reaction rates, higher yields, and better recyclability compared to traditional catalytic systems.

## 5. Recent Advances in Microwave-Assisted MCRs Using Nanoparticle Catalysts

Current developments in microwave-assisted multicomponent reactions, facilitated by nanocatalysts, focus on the remarkable synergy that helps in the one-pot, fast formation of heterocyclic molecules through an eco-friendly and green process. Nanocatalysts, especially magnetic metal oxides, carbon materials, and nanocomposites derived from them, possess high surface areas and good microwave absorbance and are easily recycled, thereby providing excellent yields in a matter of minutes. Compared to other heating methods, the reaction time is greatly reduced.

### 5.1. Synthesis of Pyridine and Pyrimidine Derivatives

Pyridine (C_5_H_5_N) is a well-known heterocyclic organic compound that forms a core structural unit in numerous natural products. Pyridine derivatives display a broad spectrum of biological activities, including antioxidant, antifungal, analgesic, antibacterial, antiglycation, antiparkinsonian, anticonvulsant, antiviral, anticancer, ulcerogenic, and anti-inflammatory effects [54,55,56]. Similarly, pyrimidine and its derivatives are pharmacologically important heterocycles with diverse therapeutic applications. They demonstrate antimalarial, antimicrobial, antidiabetic, anticancer, anthelmintic, anti-inflammatory, and anti-HIV activities, as well as roles as central nervous system depressants and cardiac agents [57,58,59].

**Scheme 1:**
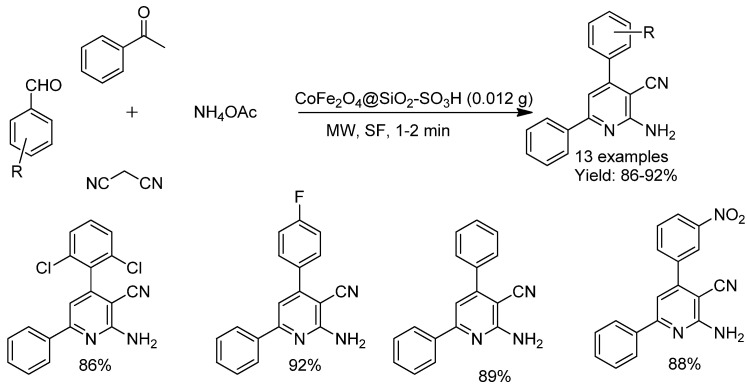
Preparation of pyridine derivatives catalysed by CoFe_2_O_4_@SiO_2_-SO_3_H NPs.

Hosseinzadeh [60] et al. (2019) reported silica-coated CoFe_2_O_4_ MNPs (CoFe_2_O_4_@SiO_2_) functionalized with chlorosulfonic acid as a competent inorganic–organic hybrid catalyst. The catalyst was employed in the microwave-aided, solvent-free synthesis of 2-amino-diarylnicotinonitrile derivatives via a 4CR of aromatic acetophenones, aldehyde analogues, malononitrile, and ammonium acetate, offering products in 86–92% yields (Scheme 1). This protocol offers numerous benefits, including short reaction times, high product yields, facile work-up, simple recrystallization for high purity, and easy catalyst recovery. Furthermore, the catalyst maintained its catalytic performance over at least five reuse cycles, demonstrating good stability and practical utility.

**Scheme 2:**
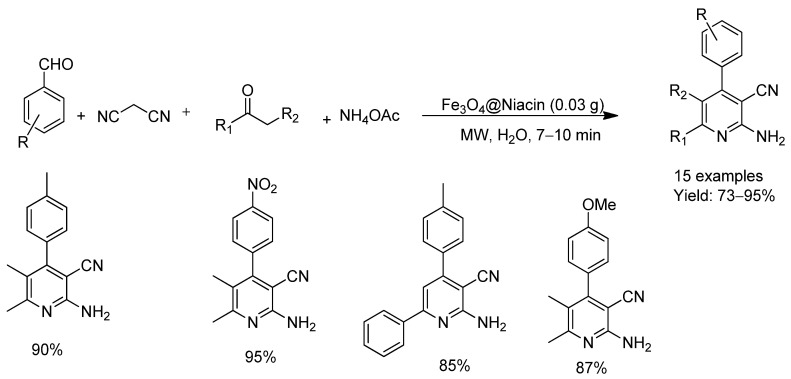
Synthesis of functionalized pyridine derivatives catalysed by MNPs-niacin.

Mojgan Afradi [61] et al. (2017) described the preparation of superparamagnetic Fe_3_O_4_ nanoparticles functionalized with vitamin B_3_ (Fe_3_O_4_@niacin), which functioned as a novel, effective, and recyclable catalyst. The synthesized Fe_3_O_4_@niacin NPs were applied as a biocatalyst for the single-pot MCRs of ketones, ammonium acetate, malononitrile, and aldehydes in water under microwave radiation, leading to the construction of cyanopyridine scaffolds. The reactions afforded good to excellent yields (73–95%), as shown in Scheme 2. The catalyst was easily retrieved and used again for six repeated runs without any major loss in catalytic action.

**Scheme 3:**
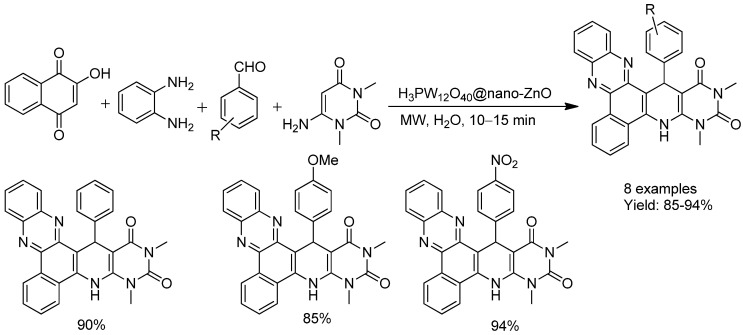
Preparation of functionalized dihydropyridine derivatives using H_3_PW_12_O_40_@nano-ZnO.

Parisa Dehghan [62] et al. (2018) established a highly competent and green procedure for the synthesis of various possibly bioactive functionalized pyrimido-phenazine derivatives via a single-pot MCR involving 6-amino-dimethyluracil, *o*-phenylenediamine, hydroxynaphthalene-dione and aromatic aldehydes. Under microwave irradiation in an aqueous medium, H_3_PW_12_O_40_@nano-ZnO functioned as a non-toxic, highly active solid acid catalyst, affording the desired products in good to excellent yields (84–92%) (Scheme 3). The catalyst demonstrated excellent recyclability and was reused for at least five repeated runs with only a minimal loss of activity.

**Scheme 4:**
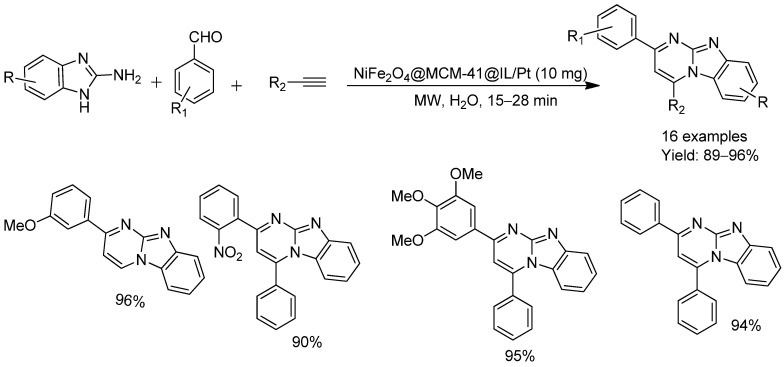
Preparation of imidazo-pyrimidine derivatives catalysed by NiFe_2_O_4_@MCM-41@IL/Pt.

Pan [63] et al. (2025) developed a novel NiFe_2_O_4_@MCM-41@IL/Pt NPs and employed it for the effective preparation of imidazo-pyrimidine derivatives via microwave-assisted A^3^ coupling reactions. The 3CR of aryl aldehydes, phenylacetylene, and 2-amino benzimidazole under microwave irradiation offered the target heterocycles in excellent yields (89–96%) within rapid reaction times (15–25 min), as illustrated in Scheme 4. The presence of a magnetic core enabled easy catalyst recovery and reuse, and the catalyst retained high catalytic activity over five successive runs without appreciable loss in efficiency.

**Scheme 5:**
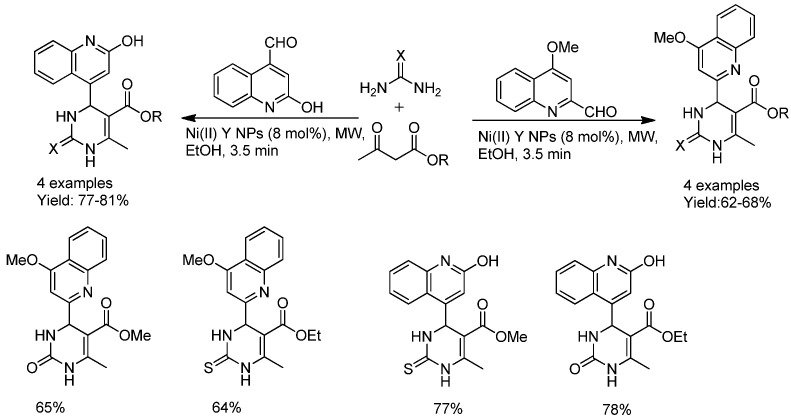
Synthesis of functionalized dihydropyrimidine derivatives catalysed by [Ni(II)Y] NPs.

Paul [64] et al. (2018) reported a microwave-assisted Ni(II)-exchanged zeolite Y [Ni(II)Y] catalyst for the Biginelli-type multicomponent synthesis of dihydropyrimidinones (DHPMs) and dihydropyrimidinethiones (DHPMTs) from quinoline aldehydes (2-hydroxy-4-formylquinoline or 2-formyl-4-methoxyquinoline), β-keto esters, and urea/thiourea (Scheme 5). The Lewis acidic Ni(II) sites promoted carbonyl activation, imine formation, and cyclocondensation, affording the desired products in 62–81% yields. The catalyst was efficiently recycled for four repeated runs without noticeable loss of activity, confirming its structural stability. The synthesized compounds exhibited notable antimicrobial activity against MRSA, *Pseudomonas aeruginosa*, and fluconazole-resistant *Candida albicans*, and demonstrated significant antioxidant scavenging activity compared to aspartic acid.

**Scheme 6:**
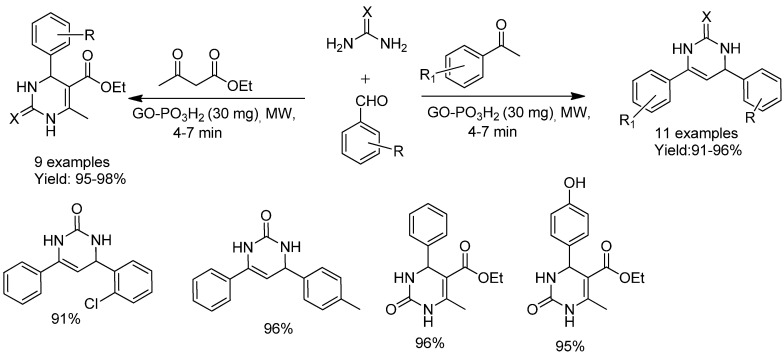
Synthesis of dihydro-pyrimidinone derivatives catalysed by PGO.

Achary [65] et al. (2018) reported a very active functionalized phosphate-graphene oxide (PGO) nanocomposite catalyst for the microwave-assisted one-pot MCRs of pyrimidinones from aromatic aldehydes, ethyl acetoacetate (or aromatic ketones), and thiourea/urea (Scheme 6). The Brønsted acidic phosphate groups promoted carbonyl activation, imine formation, and cyclocondensation, affording the desired products in 91–98% yields. The catalyst showed tremendous stability and was recycled for up to five repeated runs without substantial loss of activity.

**Scheme 7:**
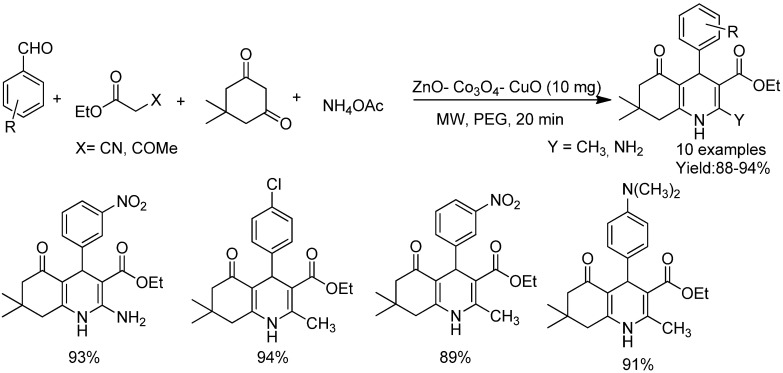
Synthesis of polyhydroquinoline derivatives catalysed by ZnO–Co_3_O_4_–CuO NPs.

Jain [66] et al. (2025) reported the preparation of a novel heterogeneous ZnO–Co_3_O_4_–CuO nanocomposite via a chemical co-precipitation method. The ternary metal oxide efficiently catalysed a microwave-assisted one-pot MCR of polyhydroquinoline derivatives from ethyl acetoacetate or ethyl cyanoacetate, various aromatic aldehydes, ammonium acetate and dimedone in PEG as a green solvent at 400 W (Scheme 7). The cooperative Lewis acidic and redox-active sites facilitated successive condensation and cyclization steps, affording products in 88–94% yields. The catalyst demonstrated good stability and was reused for up to six repeated cycles without substantial loss of activity.

**Scheme 8:**
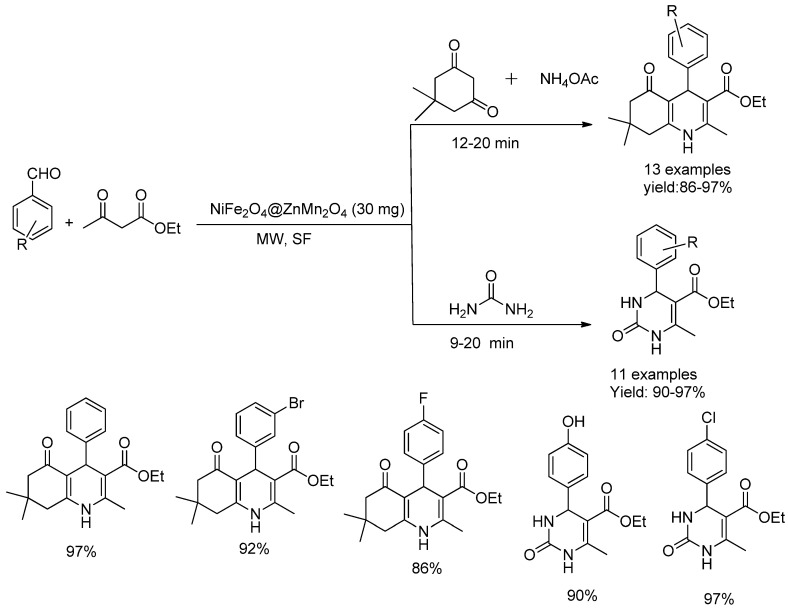
Preparation of tetrahydropyrimidine and polyhydroquinoline derivatives using NiFe_2_O_4_@ZnMn_2_O_4_ NPs.

Moradnia [67] et al. (2024) reported, for the first time, the green sol–gel synthesis of NiFe_2_O_4_@ZnMn_2_O_4_ magnetic nanocomposites (MNCs). The bifunctional nanocatalyst was employed as a heterogeneous catalyst in solvent-free, microwave-assisted MCRs for the preparation of tetrahydropyrimidines from aldehydes, ethyl acetoacetate, and urea, as well as polyhydroquinoline derivatives from aldehydes, dimedone, ammonium acetate, and ethyl acetoacetate, affording products in 86–97% yields (Scheme 8). Cooperative Lewis acidic sites facilitated the successive condensation and cyclization steps. Thanks to its magnetic properties, the catalyst could be easily retrieved with an external magnet, and it showed high catalytic effectiveness over four successive reuse cycles.

**Scheme 9:**
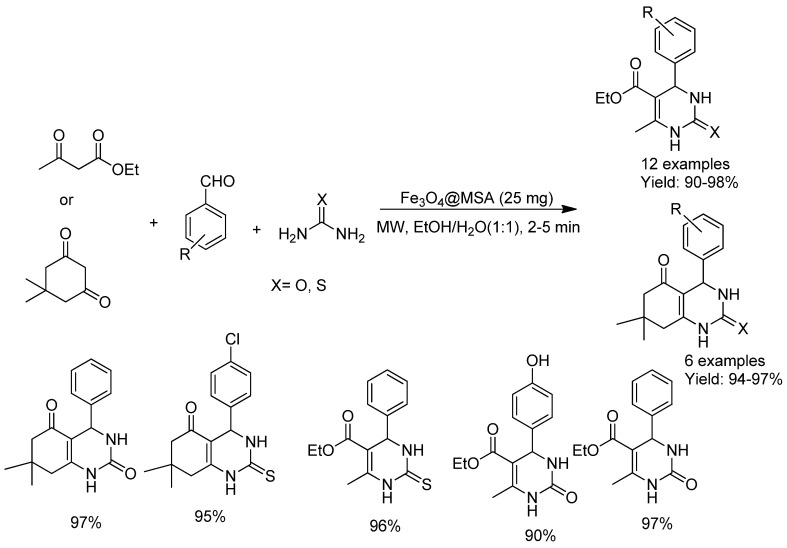
Biginelli reactions catalysed by Fe_3_O_4_@MSA.

Moradi [68] et al. (2018) reported the design and fabrication of Fe_3_O_4_@meglumine sulfonic acid as a novel, magnetically recoverable solid acid nanocatalyst. The catalyst efficiently promoted the microwave-assisted Biginelli reaction of β-dicarbonyl compounds, aldehydes, and urea/thiourea in a water/ethanol (1:1) medium, affording dihydropyrimidinone derivatives in 90–98% yields under green and rapid conditions (Scheme 9). The Brønsted acidic sulfonic sites facilitated carbonyl activation and cyclocondensation, while the magnetic core enabled easy separation and work-up. Recycling studies demonstrated that the catalyst retained high activity over four successive reuse cycles.

**Scheme 10:**
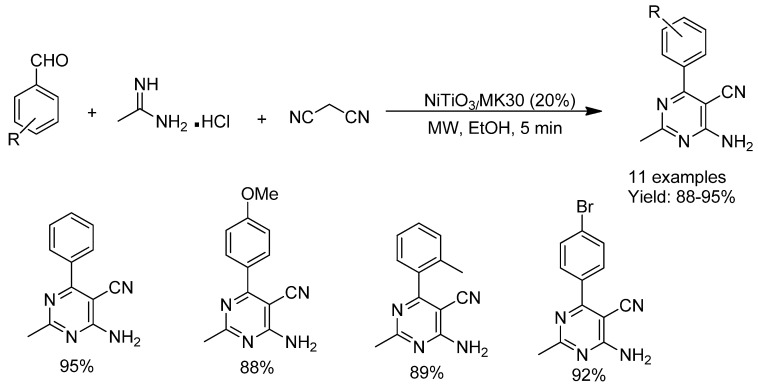
Synthesis of amino-pyrimidine derivatives catalysed by NiTiO_3_/MK30.

In 2024, Kumar [69] et al. reported an environmentally friendly synthesis of NiTiO_3_ NPs supported on exfoliated montmorillonite K30 (NiTiO_3_/MK30) via ultrasonication, which was used as a benign heterogeneous catalyst. The catalyst effectively facilitated the one-pot 3CR of 4-aminopyrimidine analogues with malononitrile, acetamidine hydrochloride and aromatic aldehydes under microwave radiation, giving excellent yields in the range of 88–95% (Scheme 10). Notably, the recovered catalyst retained good catalytic activity even after six successive cycles, indicating its stability and reusability.

**Scheme 11:**
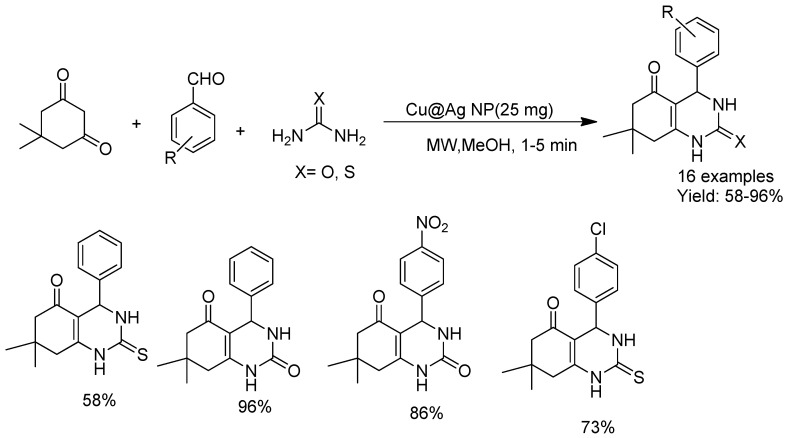
Synthesis of octahydroquinazolinones catalysed by Cu@Ag core shell.

Rout [70] et al. (2019) reported the preparation of a core–shell Cu@Ag nanocatalyst via a two-step thermal decomposition followed by a galvanic displacement reaction. The resulting core–shell nanoparticles were effectively employed as a catalyst for the one-pot multicomponent synthesis of octahydroquinazolinone derivatives from dimedone, aromatic aldehydes, and urea under microwave irradiation in methanol, affording products in 58–96% yields (Scheme 11). Furthermore, the catalyst demonstrated competent stability and could be reused for up to five consecutive cycles without substantial loss of catalytic activity.

**Scheme 12:**
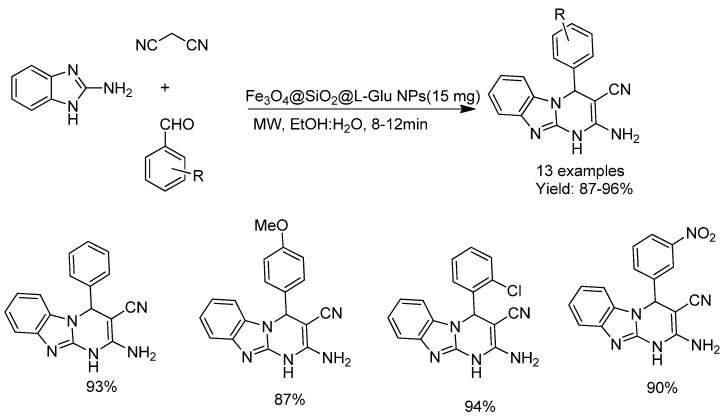
Synthesis of benzo-imidazo-pyrimidine derivatives catalysed byFe_3_O_4_@SiO_2_@L-glutamine.

Thongni [71] et al. (2024) reported the MNPs functionalized using L-glutamine (Fe_3_O_4_@SiO_2_@L-glutamine NPs) as an efficient heterogeneous catalyst. The catalyst promoted the single-step preparation of benzo-imidazo-pyrimidines from 2-aminobenzimidazole, benzaldehyde, and malononitrile under microwave radiation in water, delivering 87–96% yields (Scheme 12). Notably, the catalyst retained reasonable activity over five repeated runs, with no considerable loss in catalytic execution.

**Scheme 13:**
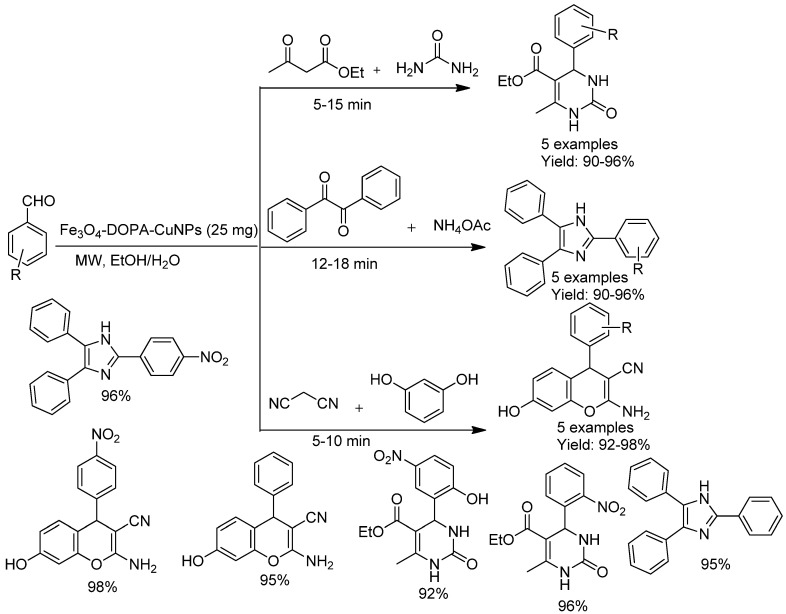
Synthesis of dihydropyrimidinones, imidazole and pyran derivatives catalysed by Fe_3_O_4_-DOPA-Cu NPs.

Kumari [72] et al. (2019) reported magnetically detachable copper-loaded L-DOPA-functionalized MNPs (Fe_3_O_4_–DOPA–Cu NPs) as a versatile heterogeneous catalyst. This single catalyst exhibited excellent catalytic activity in multiple microwave-aided MCRs (Scheme 13), including: (i) the Biginelli synthesis of dihydropyrimidinones (DHPMs) from aldehydes, ethyl acetoacetate, and urea; (ii) imidazoles from benzil, ammonium acetate and aromatic aldehydes; and (iii) chromenes from resorcinol, malononitrile, and aromatic aldehydes. Notably, the catalyst was easily recovered magnetically and reused for several cycles in all reactions without substantial loss of catalytic action, demonstrating its robustness and reusability.

**Scheme 14:**
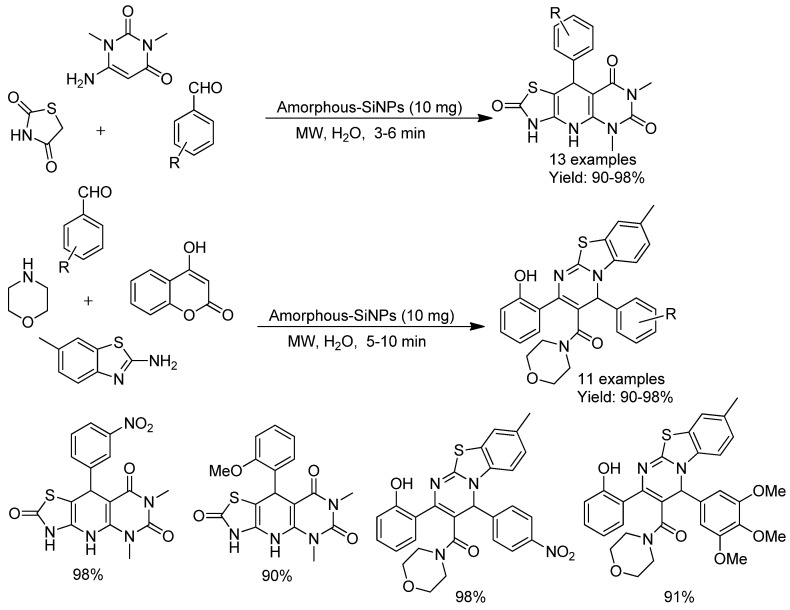
Synthesis of pyrido-pyrimidine derivatives and thiazolopyrimidine derivatives.

Alshahrani [73] et al., 2025 reported the preparation of amorphous silica NPs (amorphous-SiNPs) obtained from rice husk ash, which were thoroughly characterized using various physicochemical methods. The synthesized SiNPs were then successfully employed as efficient heterogeneous catalysts for the rapid microwave-assisted synthesis of pyrido pyrimidine derivatives via a MCR involving benzaldehyde, 2,4-thiazolidinedione, *N*,*N*-dimethyl-6-aminouracil, and in aqueous media. In addition, the catalyst exhibited excellent activity in the production of thiazolopyrimidine derivatives through the reaction of benzaldehyde, 4-hydroxycoumarin, morpholine, and 2-amino-6-methylbenzothiazole under microwave irradiation in water, affording the desired products in high yields of 90–98% (Scheme 14). Notably, the catalyst retained its effectiveness over nine successive cycles with only negligible loss of activity.

### 5.2. Microwave-Assisted Synthesis of Imidazole and Indazole Derivatives

Imidazoles are nitrogen-containing heterocycles that have gained considerable attention because of their wide range of biological and pharmacological activities. They act as important scaffolds in the design of bioactive compounds, including antiviral, anticancer, antiaging, anticoagulant, anti-inflammatory, antibacterial, antitubercular, antidiabetic, and antimalarial agents, as well as enzyme inhibitors [74,75,76]. Similarly, the indazole ring is one of the most extensively used structural frameworks in medicinal chemistry. Several substituted indazoles display significant biological activities, making them promising candidates for the development of anticancer agents [77,78].

**Scheme 15:**
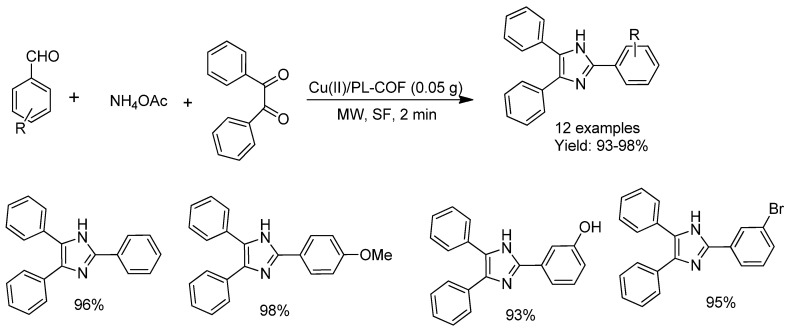
Preparation of triaryl imidazole derivatives using Cu(II) PL-COF.

Sedaghat [79] et al. (2023) described the fabrication of copper(II)/polyimide-linked covalent organic frameworks (Cu(II)/PL-COFs) and demonstrated their effectiveness as heterogeneous catalysts for the one-pot synthesis of trisubstituted imidazole derivatives. The reactions proceeded via a 3CR of various aldehydes, ammonium acetate and benzil under solvent-less conditions using microwave irradiation, giving the target products in excellent yields ranging from 93 to 98%, as illustrated in Scheme 15. The catalyst showed high reusability and was successfully recovered for up to five successive cycles without a significant loss of catalytic activity.

**Scheme 16:**
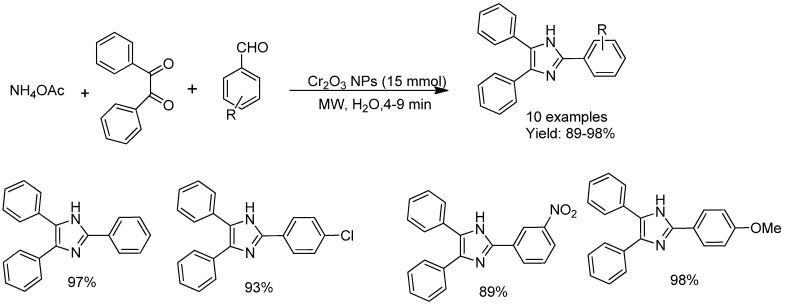
Synthesis of imidazole derivatives using Cr_2_O_3_ NPs.

Ahmadi [80] et al. (2022) reported a simple, cost-effective, and eco-friendly biological approach for the creation of Cr_2_O_3_ NPs using Cr(NO_3_)_3_·9H_2_O as the precursor and *Zingiber officinale* extract as a natural reducing and stabilizing agent. The biosynthesized Cr_2_O_3_ NPs were utilized as an effective Lewis acid catalyst for the synthesis of imidazole via the condensation of ammonium acetate, benzil, and aromatic aldehydes. The reactions were carried out in water as a green solvent under microwave radiation, providing the target imidazole in excellent yields (89–98%), as illustrated in Scheme 16. Notably, the Cr_2_O_3_ could be easily recovered and reclaimed, maintaining high catalytic efficacy even after six successive runs. Similarly, green-synthesised Cr_2_O_3_ NPs derived from agricultural waste extracts have demonstrated excellent catalytic activity, structural stability, and recyclability, supporting their use as sustainable nanocatalysts in organic synthesis.

**Scheme 17:**
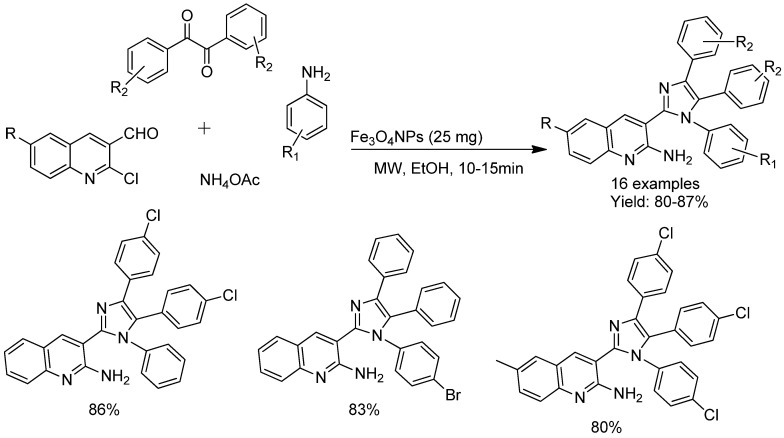
Preparation of triaryl imidazolyl quinoline derivatives catalysed by Fe_3_O_4_ NPs.

Bheemayya [81] et al. (2025) reported a Fe_3_O_4_ nanoparticle-catalysed cascade multicomponent protocol for the microwave-assisted synthesis of quinoline-functionalized imidazole derivatives from 2-chloroquinoline-3-carbaldehydes, anilines, and substituted benzil (Scheme 17). The surface Lewis acidic sites of Fe_3_O_4_ facilitated the sequential condensation, cyclization, and aromatization steps, affording the target compounds in 80–87% yields. The synthesized derivatives exhibited significant inhibitory action against COX-1 and/or COX-2 enzymes, along with notable antioxidant activity.

**Scheme 18:**
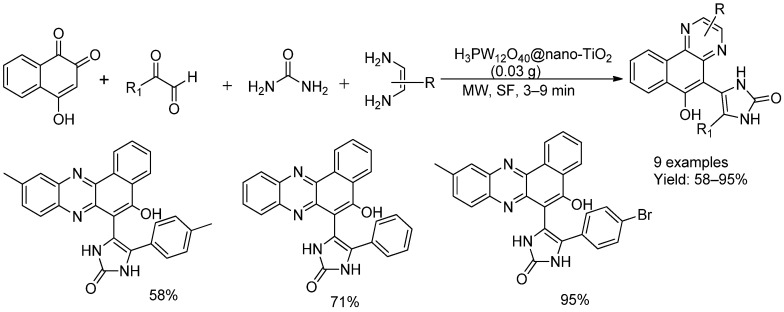
Synthesis of benzo-phenazinyl imidazolone derivatives catalysed by H_3_PW_12_O_40_@nano-TiO_2_.

Taheri [82] et al. (2022) reported H_3_PW_12_O_40_@nano-TiO_2_ as a reusable heterogeneous catalyst for carrying out MCRs. This catalyst proved to be an effective protocol for synthesizing benzo-phenazinyl-imidazolone derivatives through the condensation of benzene-1,2-diamine, arylglyoxals, urea, and 2-hydroxynaphthalene-1,4-dione under microwave irradiation (Scheme 18). The products were obtained within a yield range of 58 to 95%. The catalyst exhibited good recyclability and was reused for up to six consecutive cycles under identical reaction conditions without any substantial loss in catalytic performance.

**Scheme 19:**
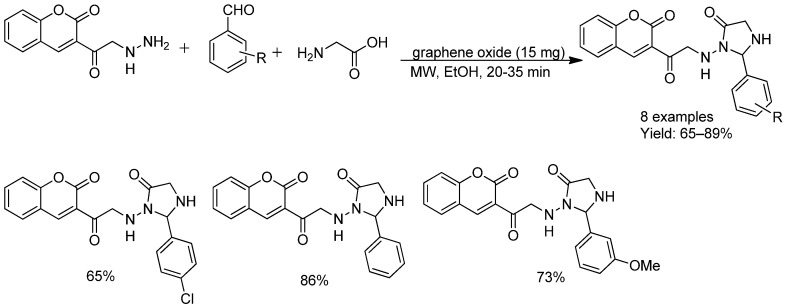
Synthesis of chromene-functionalized imidazolidinone derivatives catalysed by graphene oxide.

Radhi [83] et al. (2022) reported an efficient one-pot 3CR protocol for the preparation of chromene-incorporated imidazolidinone derivatives using graphene oxide nanosheets as a catalyst. The reaction involved 3-(2-hydrazinylacetyl)-2*H*-chromen-2-one, various aromatic aldehydes, and glycine under microwave radiation, affording the desired products in good yields (65–89%), as illustrated in Scheme 19. The graphene oxide nanosheets showed superb stability and reusability, retaining their catalytic activity for up to seven repeated reaction series without any noticeable loss in efficiency.

**Scheme 20:**
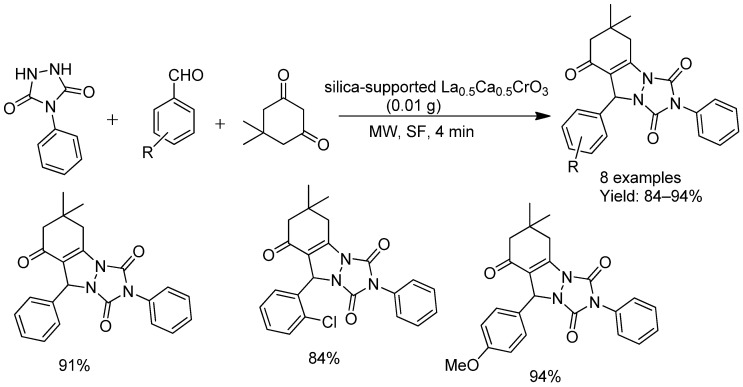
Synthesis of triazolo-indazole-triones catalysed by LCCO or S-LCCO NPs.

Najmeh Zahedi [84] et al. (2018) reported the preparation of novel perovskite oxide NPs and their function as efficient catalysts for the preparation of triazolo-indazole-trione derivatives. The compounds were synthesized via a three-component cyclocondensation reaction of urazole, aldehydes, and dimedone under microwave radiation and solvent-less conditions, as shown in Scheme 20. The protocol afforded the desired compounds in good to excellent yields (84–94%). Both La_0.5_Ca_0.5_CrO_3_ and silica-supported La_0.5_Ca_0.5_CrO_3_ functioned as reusable acidic solid catalysts, retaining high catalytic action for at least four repeated runs without any significant loss in efficiency.

### 5.3. Microwave-Assisted Synthesis of Pyran and Chromene Derivatives

Oxygen-containing heterocyclic frameworks are widely employed in organic synthesis due to their significant medicinal importance. Among these, pyran ring systems have attracted considerable attention because of their broad spectrum of therapeutic activities, including antidiabetic, antibacterial, antitubercular, anticancer, anti-HIV, and antiproliferative effects [85,86,87]. When a pyran ring is fused with a benzene ring at the 5,6-positions, it forms the 1-benzopyran (chromene) scaffold, a bicyclic oxygen-containing heterocycle. Chromene derivatives also exhibit diverse biological properties such as antioxidant, anticancer, anti-inflammatory, anticonvulsant, antitubercular, and antibacterial activities [88,89,90].

**Scheme 21:**
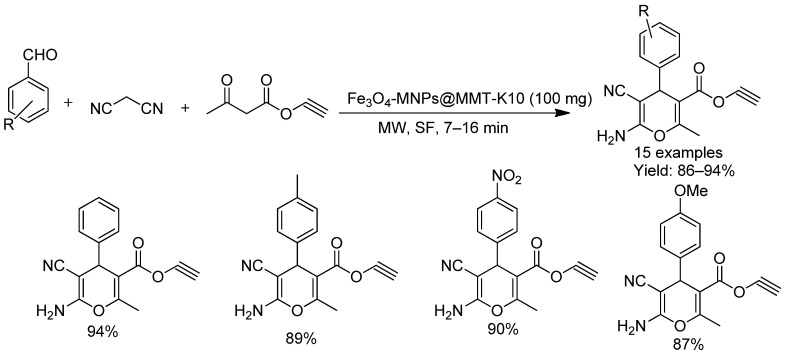
Synthesis of propargyl-substituted pyran derivatives using catalytic activity of Fe_3_O_4_-MNPs@MMT-K10.

Thanh [91] et al. (2023) described recoverable heterogeneous MNPs, Fe_3_O_4_-MNPs@MMT-K10, which were successfully used for the one-pot microwave-aided preparation under solvent-free conditions of highly substituted pyran and chromene derivatives bearing a propargyl unit (Scheme 21). The reaction involved a 3CR of 3-propargyloxyphenol/propargyl acetoacetate, arylaldehydes and malononitrile affording products in 86–94% yields. The catalyst demonstrated excellent strength and could be reused at least six times without substantial loss of catalytic activity.

**Scheme 22:**
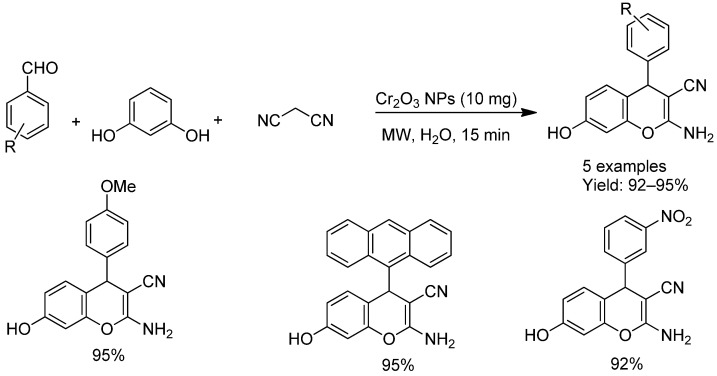
Synthesis of chromene derivatives catalysed by Cr_2_O_3_ NPs.

Okram [92] et al. (2025) reported the green preparation of chromium (III) oxide (Cr_2_O_3_) NPs using *Hamelia patens* leaf extract. The biosynthesized NPs were effectively employed in a one-pot preparation of chromene derivatives (Scheme 22) from resorcinol, malononitrile, and benzaldehyde under microwave irradiation in water, affording excellent yields (92–95%). In addition, the antioxidant activity of the chromene products was assessed using the DPPH test in ethyl acetate, methanol and acetone, revealing significant solvent-reliant variations in radical scavenging action.

**Scheme 23:**
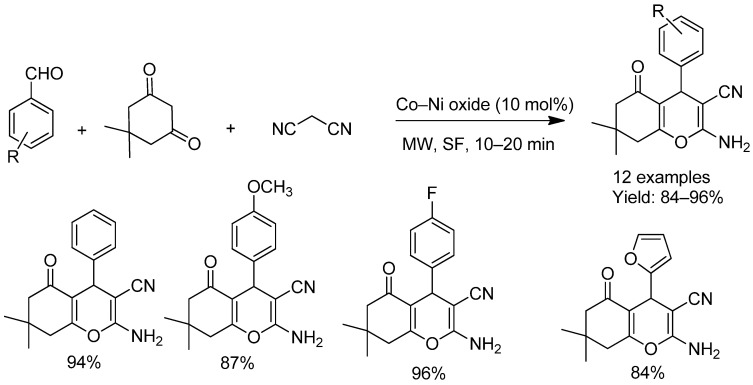
Synthesis of benzo-pyran derivatives catalysed by Co–Ni mixed oxide NPs.

Jopale [93] et al. (2024) reported the green protocol for a Co–Ni mixed oxide catalyst made from cheap precursors using Euphorbia latex as a bio-based solvent. The mixed metal oxide efficiently catalysed a microwave-assisted condensation of malononitrile and various aromatic aldehydes with dimedone to afford tetrahydro-benzo-pyran derivatives in 84–96% yields (Scheme 23). Notably, the catalyst showed outstanding stability and was recyclable for up to 12 continuous cycles with no significant diminution of activity, demonstrating its sustainability.

**Scheme 24:**
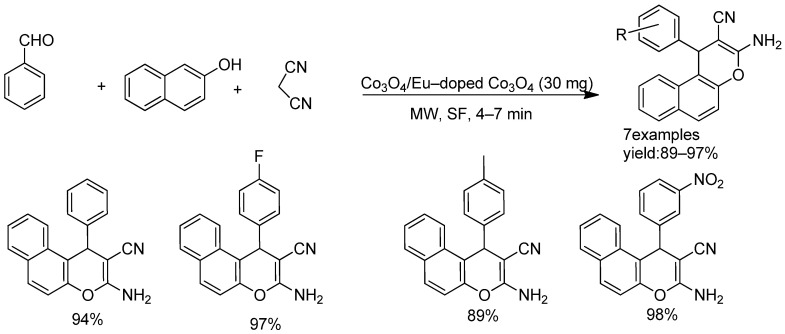
Preparation of benzochromenes catalysed by Co_3_O_4_ and Eu-doped Co_3_O_4_ NPs.

Ahmadi [94] et al. (2021) reported the ultrasonic-supported preparation of Co_3_O_4_ and Eu-doped Co_3_O_4_ nanocatalysts and evaluated their catalytic efficiency in the solvent-free, microwave-assisted multicomponent preparation of 2-amino-benzochromenes from malononitrile, aromatic aldehydes, and β-naphthol (Scheme 24). The metal oxide surfaces promoted subsequent Knoevenagel condensation, Michael addition, and cyclization, affording products in 89–97% yields. The catalysts were readily recovered by filtration, washed with hot ethanol, and dried, maintaining high catalytic activity over six repeated runs without substantial loss of performance.

**Scheme 25:**
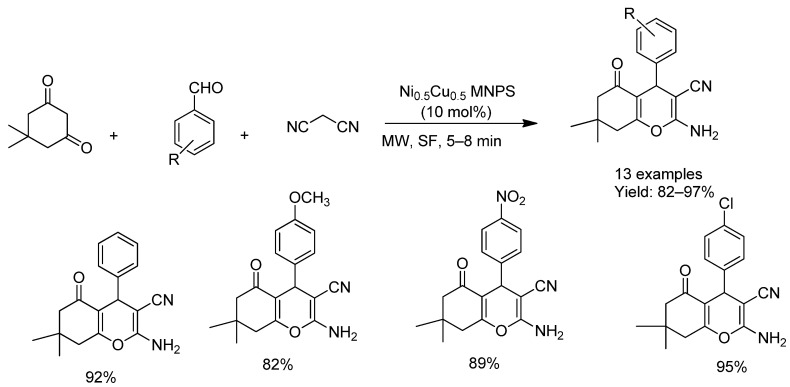
Synthesis of tetrahydropyran using Ni_0.5_Cu_0.5_Fe_2_O_4_ catalyst.

Ahankar [95] et al. (2020) revealed the preparation of Ni_0.5_Cu_0.5_Fe_2_O_4_ magnetic nanoparticles by a sol gel process mediated by Arabic gum (AG), which acts as a green reducing agent. Furthermore, Ni_0.5_Cu_0.5_Fe_2_O_4_ NPs were used for the one-pot, multicomponent preparation of tetrahydro-benzo-pyrans under microwave radiation conditions without the requirement for any solvent. The condensation of dimedone, malononitrile, and various aldehydes gave the preferred products in high yield (82–97%) as shown in Scheme 25. After each reaction, the catalyst was magnetically separated, washed with ethanol, and reused for five cycles without loss of activity.

**Scheme 26:**
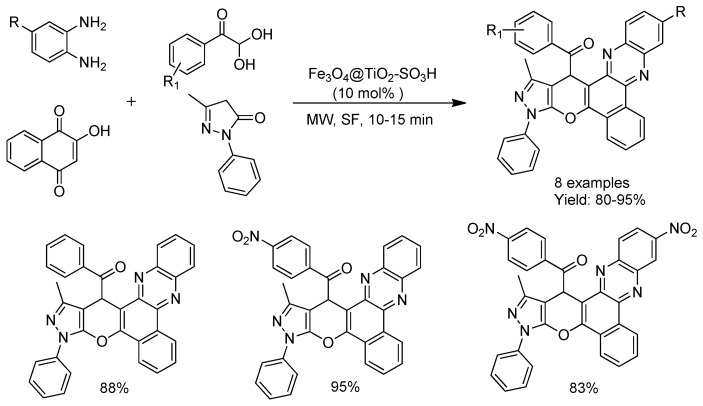
Synthesis of pyrazolo-pyran derivatives catalysed by Fe_3_O_4_@TiO_2_–SO_3_H NPs.

Taheri [96] et al. (2020) developed a recyclable Fe_3_O_4_@TiO_2_–SO_3_H nanocatalyst for microwave-assisted one-pot MCRs of functionalized pyrazolo-pyran derivatives (Scheme 26). The Brønsted acidic –SO_3_H sites facilitated the sequential condensation of o-phenylenediamine, 2-hydroxynaphthalene-1,4-dione, 5-methyl-2-phenyl-3*H*-pyrazol-3-one and arylglyoxals, affording products in 80–95% yields. The magnetically recoverable catalyst retained its activity over three reuse cycles. The synthesized compounds exhibited poor to moderate antibacterial activity against the tested strains.

### 5.4. Microwave-Assisted Synthesis of Propargylamine Derivatives

Propargylamines are highly versatile building blocks in organic synthesis. Their structural motif occurs in numerous natural products, phytoprotective agents, pharmaceuticals, and other biologically important compounds [97,98].

**Scheme 27:**
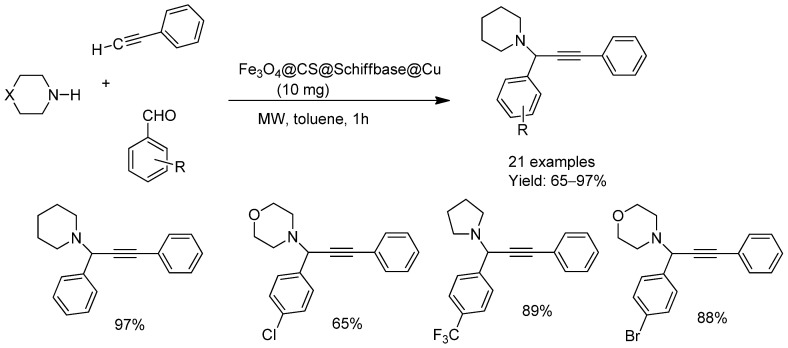
Fe_3_O_4_@CS@Schiff base@Cu effectively catalysed the formation of propargylamine derivatives.

Hasan [99] et al. (2023) have also demonstrated the successful construction of a new heterogeneous catalyst, Fe_3_O_4_@CS@Schiff base@Cu, through the immobilization of a Cu(II) Schiff base complex on a Fe_3_O_4_–chitosan matrix. The prepared heterogeneous catalyst displayed high catalytic action towards the A^3^ coupling reaction of aldehydes, amines, and alkynes to synthesize propargylamine in good to excellent yields (65–97%) under microwave radiation, as shown in Scheme 27. This catalyst possessed outstanding recyclability and could preserve its catalytic efficiency at around 95% even after conducting six consecutive reactions.

**Scheme 28:**
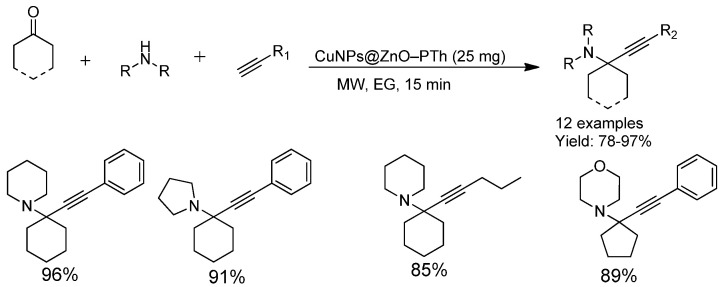
Synthesis of tetra-substituted propargylamine catalysed by CuNPs@ZnO–PTh.

Shah [100] et al. (2018) reported the preparation of Cu nanoparticles supported on a ZnO–polythiophene (ZnO–PTh) nanocomposite (CuNPs@ZnO–PTh) via a simple impregnation method. The heterogeneous catalyst efficiently promoted the microwave-assisted preparation of propargylamines in ethylene glycol as a recyclable and eco-friendly solvent, affording products in 78–97% yields (Scheme 28). The extreme surface area and synergistic interaction between CuNPs and the ZnO–PTh support facilitated effective C–N bond formation, enabling a broad substrate scope under mild conditions. The catalyst exhibited short reaction times, easy work-up, and good recyclability without substantial loss of activity, highlighting its green and sustainable nature.

**Scheme 29:**
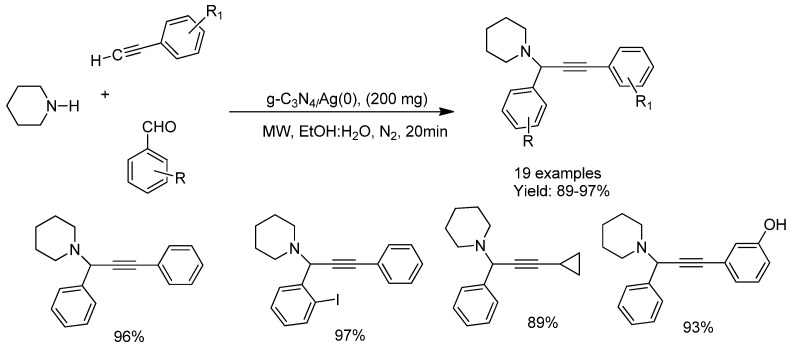
Preparation of propargylamines catalysed by AgNPs@g-C_3_N_4._

In 2018, Patel [101] and co-workers produced silver nanoparticles supported on graphitic carbon nitride (AgNPs@g-C_3_N_4_) and employed them as an effective heterogeneous catalyst for the A^3^ coupling reaction of secondary amines, aldehydes, and terminal alkynes to afford propargylamines in excellent yields (89–97%) under microwave irradiation in ethanol (Scheme 29). Notably, no significant loss in catalytic activity was observed even after six successive recycling cycles, confirming the robustness of the catalyst.

### 5.5. Microwave-Assisted Synthesis of Phenazine Derivatives

Fused benzene moieties are found at the carbon positions of a pyrazine nucleus in phenazine heterocycles. One important class of aza-polycyclic compounds is phenazine. Phenazine systems have indispensable advantages over other nitrogen heterocyclic compounds, including strong and stable fluorescence emission, superior biocompatibility, and excellent antibacterial and antifungal properties [102,103,104].

**Scheme 30:**
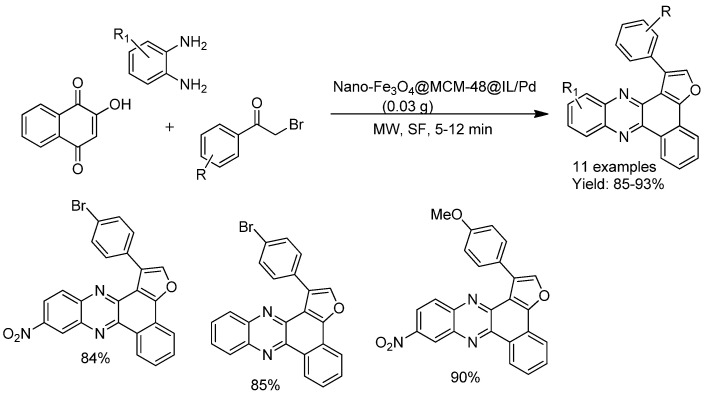
Synthsis of benzo-furo-phenazines catalysed by Fe_3_O_4_@MCM-48@IL/Pd.

Taheri [105] et al. (2024) reported a new mesoporous Fe_3_O_4_@MCM-48@IL/Pd catalyst that was successfully employed as a reusable heterogeneous catalyst for the four-constituent, microwave-aided preparation of benzo-furo-phenazine derivatives. The reaction involved *o*-phenylenediamine, 2-hydroxynaphthalene-1,4-dione, and *p*-bromo phenacyl bromide under solvent-free conditions using microwave radiation, affording the desired products in excellent yields of 85–93% (Scheme 30). Notably, the magnetic catalyst could be recovered and reused for up to six consecutive cycles without substantial loss of catalytic action. In addition, the catalyst revealed effective functioning in the degradation of rhodamine B under optimized conditions.

**Scheme 31:**
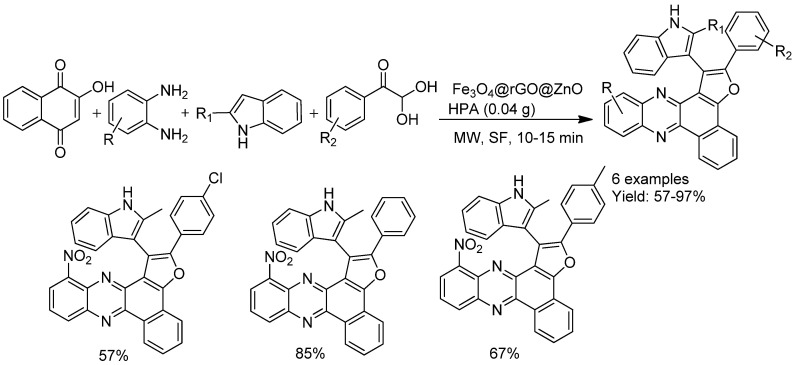
Preparation of benzo-furo-phenazine derivatives catalysed by Fe_3_O_4_@rGO@ZnO–HPA.

Fe_3_O_4_@rGO@ZnO–HPA MCNPs were prepared by Taheri [106] et al. in 2023 as an efficient heterogeneous catalyst. This catalyst was effectively utilized for the microwave-aided, solvent-free preparation of benzo-furo-phenazine derivatives via the reaction of arylglyoxals, benzo-phenazinol, and methylindole, providing products in yields ranging from 57 to 97% (Scheme 31). Interestingly, the catalyst could be recovered and recycled for several cycles with no appreciable loss of catalytic action.

**Scheme 32:**
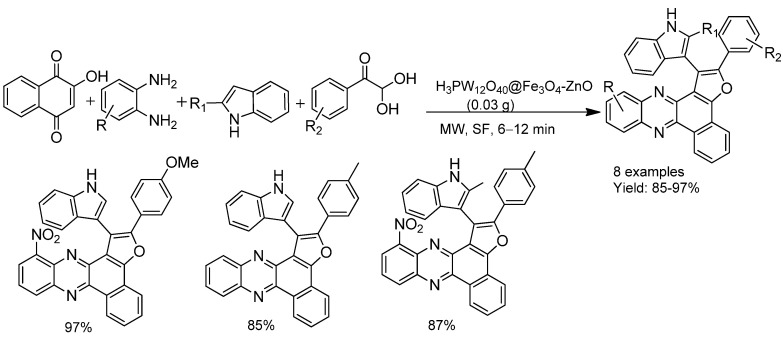
Synthesis of benzo-furo-phenazine derivatives using H_3_PW_12_O_40_@Fe_3_O_4_/ZnO NPs.

Taheri [107] et al. (2021) described an efficient nanocatalyst, H_3_PW_12_O_40_@Fe_3_O_4_/ZnO, for the preparation of furo-phenazine derivatives. The catalyst was successfully applied in a 3CR between orthophenylene diamine, indoles, 2-hydroxy-naphthalene-1,4-dione and arylglyoxals, affording products in excellent yields ranging from 85 to 97%, as illustrated in Scheme 32. The nanocatalyst showed good recyclability and could be reused for six successive runs without any major loss in catalytic action. The reaction protocol has some merits, including the use of solvent-free conditions under microwave irradiation and starting materials without any prior activation or modifications. Apart from its synthetic application, the catalyst also showed very good photocatalytic activity, degrading methylene blue by up to 97% under optimized conditions.

### 5.6. Microwave-Assisted Synthesis of Quinazolinone and Quinoxaline Derivatives

Quinazoline and quinazolinone are among the most significant nitrogen-containing heterocycles in medicinal chemistry, exhibiting a wide array of biological activities such as analgesic, antifungal, antibacterial, anti-inflammatory, anticonvulsant, anticancer and anti-HIV effects [108,109]. Quinoxaline is another important nitrogenous heterocyclic scaffold extensively used in medicinal chemistry. It displays a remarkably broad spectrum of biological properties, including antibacterial, anticancer, anticonvulsant, anti-inflammatory, antifungal, antioxidant, antitubercular, antiprotozoal, antiviral, and antidiabetic activities [110,111,112].

**Scheme 33:**
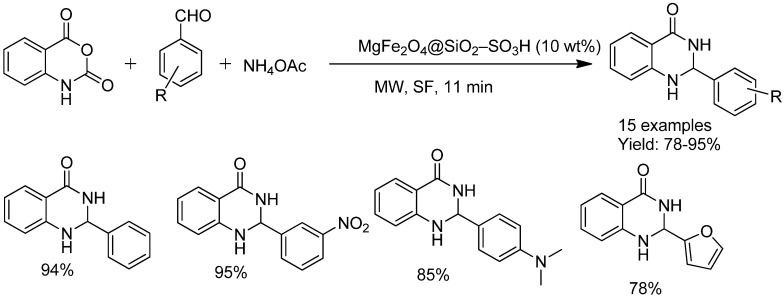
Synthesis of quinazolinone derivatives using MgFe_2_O_4_@SiO_2_–SO_3_H.

Aswar [113] et al. (2021) investigated the catalytic functioning of a recoverable MgFe_2_O_4_@SiO_2_–SO_3_H nanocatalyst for the preparation of dihydroquinazolinone derivatives. The reaction was carried out via an MCR of isatoic anhydride, ammonium acetate, and aldehydes under solvent-free microwave conditions, providing the target products in good to excellent yields (78–95%) (Scheme 33). The catalyst was easily retrieved using an external magnet and effectively reused for up to five successive reaction cycles under identical conditions without loss of catalytic activity.

**Scheme 34:**
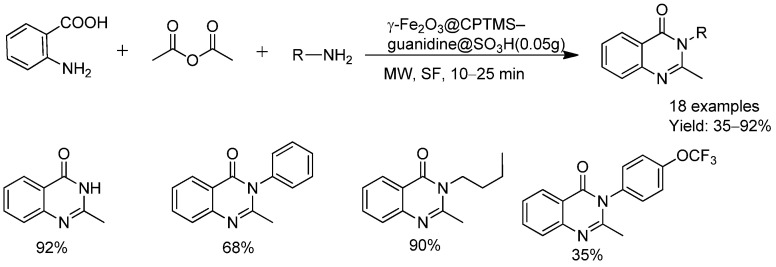
Preparation of quinazolin-4(3H)-one derivatives.

Norouzi [114] et al. (2021) reported the preparation of a new organic–inorganic nanohybrid, γ-Fe_2_O_3_@CPTMS–guanidine@SO_3_H, functionalized with sulfonic acid groups. This nanocatalyst was effectively employed in the one-pot preparation of quinazolinone derivatives via the condensation of various amines, anthranilic acid and acetic anhydride under microwave radiation and solvent-less conditions (Scheme 34). The protocol afforded the required products in modest to exceptional yields (35–92%). Magnetic decantation with an external magnet enabled efficient separation of the catalyst, which was reused for six successive runs while retaining its catalytic efficiency.

**Scheme 35:**
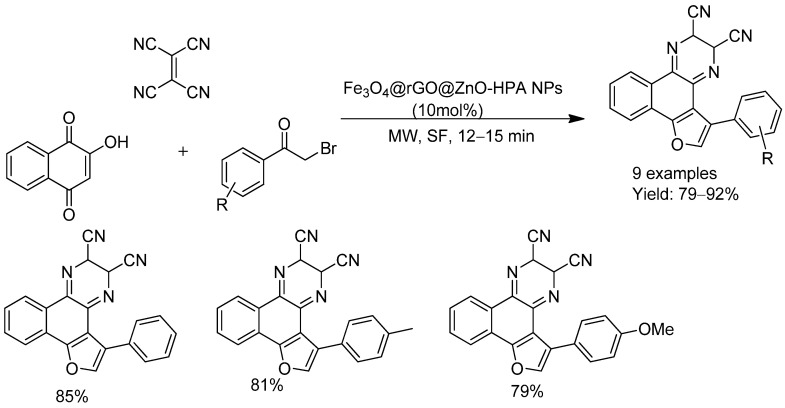
Synthesis of benzo-furo-quinoxaline derivatives catalysed by Fe_3_O_4_@rGO@ZnO–HPA.

Taheri [115] et al. (2023) reported a magnetic core–shell NP catalyst (Fe_3_O_4_@rGO@ZnO–HPA, MCNPs) as a reusable solid catalyst. The catalyst effectively facilitated the one-pot, 4CR preparation of benzo-furo-quinoxaline derivatives from tetracyanoethene, 2-hydroxynaphthalene-1,4-dione, and phenacyl bromides under microwave-supported, solvent-free conditions, affording products in 79–92% yields (Scheme 35). In addition, the synthesized materials were applied to the degradation of methylene blue, demonstrating potential for the treatment of organic dye pollutants. The catalyst could be reused for up to six repeated cycles with only a slight loss of catalytic activity, highlighting its robustness and practical applicability.

### 5.7. Microwave-Assisted Synthesis of Xanthene Derivatives

Xanthenes represent a significant class of heterocyclic compounds in medicinal chemistry, owing to their wide-ranging pharmacological activities. Structurally characterized by a dibenzo-pyran core with a central oxygen atom, these tricyclic aromatic compounds have broad applications in pharmaceuticals as well as in the food, textile, dye, electro-optical, and bioimaging fields. Xanthene derivatives exhibit notable biological activities such as antiparasitic, antileishmanial, antibacterial, neuroprotective, cytotoxic, and photophysical effects, making them useful in drug discovery [116,117,118].

**Scheme 36:**
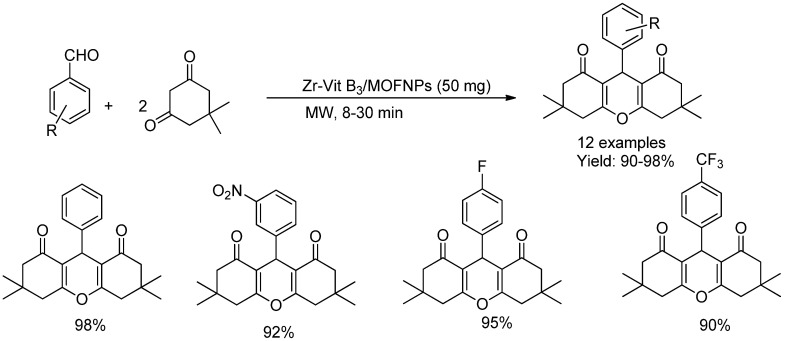
Preparation of xanthene derivatives using Zr-MOF.

A new zirconium/vitamin B3 (Zr/VitB3) metal–organic framework (Zr-MOF) was designed, and its catalytic activity was investigated in organic synthesis (Alsalhi [119] et al. 2025). The prepared Zr-MOF showed remarkable catalytic activity for the preparation of tetrahydroxanthene derivatives in moderate to high yields (58–95%) from the condensation of dimedone and various benzaldehydes under microwave radiation, as depicted in Scheme 36. The reusability of the catalyst was also examined, showing that the Zr-MOF could be used for at least three cycles with negligible loss of activity. Furthermore, molecular docking analyses showed that the synthesized xanthene derivatives had higher binding affinity to HIV-related targets than the standard drug, suggesting their potential to act as effective anti-HIV agents.

**Scheme 37:**
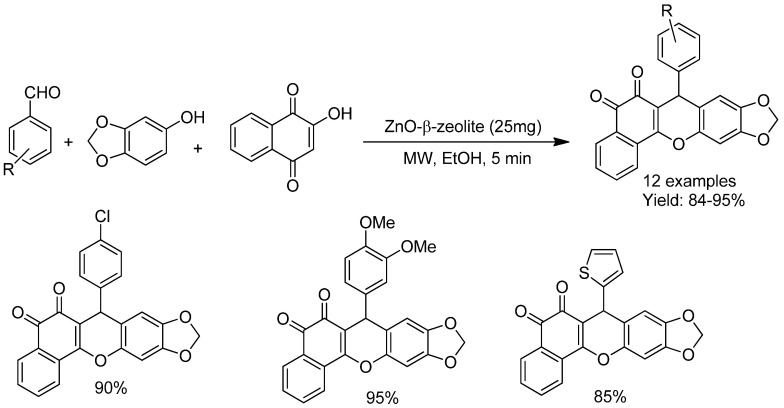
Synthesis of benzodioxolo-xanthenone derivatives catalysed by ZnO–β-zeolite.

Lambat [120] et al. (2020) reported ZnO–β-zeolite nanoparticles as an inexpensive and effective heterogeneous catalyst. This catalyst was effectively applied in the one-pot MCR of benzodioxolo-xanthenedione derivatives from benzaldehyde, 2-hydroxy-1,4-naphthoquinone and 3,4-methylenedioxyphenol under microwave radiation in ethanol, affording products in 84–95% yields (Scheme 37). The recovered catalyst exhibited comparable catalytic efficiency to the fresh catalyst and could be reused for up to four cycles, with only a slight decrease in yield attributed to minor catalyst loss during recovery. Overall, the method offers several advantages, including high product yields, simple work-up, short reaction times, microwave-assisted energy efficiency, and good catalyst recyclability.

### 5.8. Microwave-Assisted Synthesis of Acridine Derivatives

Acridine and acridone derivatives are nitrogen-containing heterocycles known for their diverse medicinal properties. Their distinct physicochemical characteristics, broad biological activities, and industrial relevance make acridine derivatives particularly significant. These compounds also exhibit a range of biological activities, including anti-inflammatory, anticancer, and antibacterial effects [121,122,123].

**Scheme 38:**
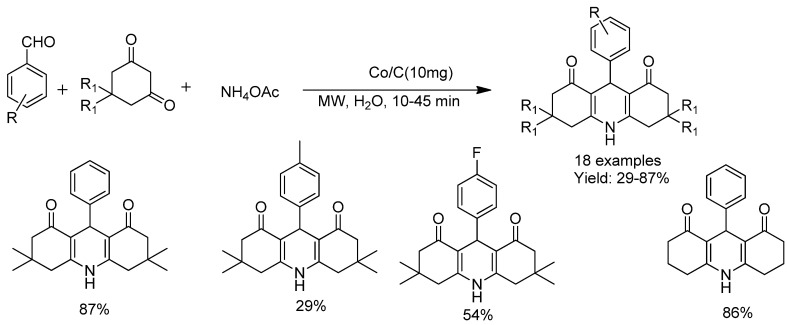
Preparation of hexahydroacridine-dione derivatives using Co/C NPS.

Nguyen [124] et al. (2024) reported the synthesis of a Co/C nanocatalyst possessing Lewis acidic sites, which functioned as a competent and green catalyst for a microwave-assisted MCR of dimedone, benzaldehyde and ammonium acetate to afford the corresponding heterocyclic products (Scheme 38). The Lewis acid sites facilitated carbonyl activation and subsequent condensation–cyclization steps, delivering products in 29–87% yields. Under optimized conditions, the catalyst showed good stability and was successfully recycled for up to five successive runs without substantial loss of catalytic efficacy.

### 5.9. Microwave-Assisted Synthesis of Azlactone Derivatives

Azlactones (oxazolones) are versatile intermediates extensively employed in organic synthesis. They exhibit a broad spectrum of biological activities, including antifungal, anti-inflammatory, and antibacterial properties, and serve as key precursors in the synthesis of penicillin-type therapeutics and synthetic hormone derivatives [125,126,127].

**Scheme 39:**
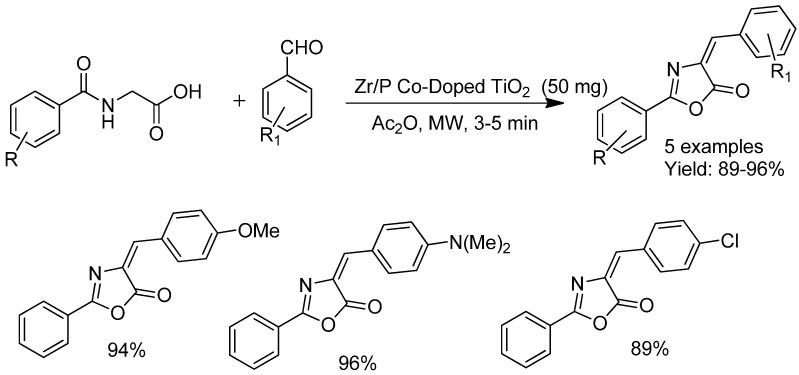
Synthesis of azlactones catalysed by Zr/P co-doped TiO_2_.

Buddiga [128] et al. (2026) described a new Zr/P co-doped TiO_2_ nanocatalyst synthesized via the sol-gel method. The catalyst was efficiently applied to the single-step microwave-supported preparation of azlactones from hippuric acid and aromatic aldehydes, affording products in 89–96% yields (Scheme 39). This procedure remarkably reduces reaction times while increasing yields, thus providing a more efficient and greener alternative to previously developed methods.

### 5.10. Microwave-Assisted Synthesis of Thiophene Derivatives

Thiophene is a five-membered heteroaromatic ring system featuring a sulphur atom at the 1-position. Thiophene derivatives demonstrate varied biological activities, including anticancer, antibacterial, analgesic, anti-inflammatory, and antihypertensive properties, as well as applications in the fabrication of light-emitting diodes [129,130,131].

**Scheme 40:**
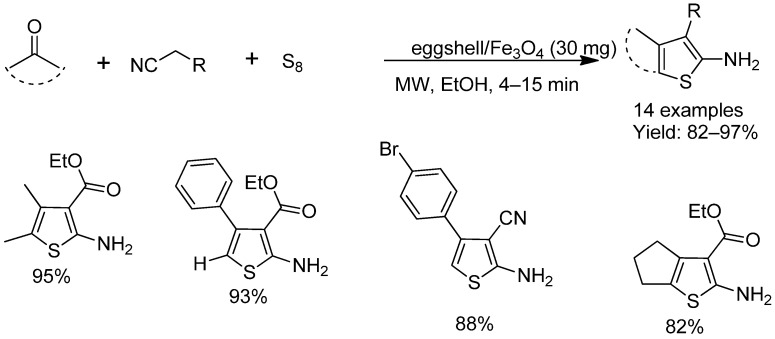
Synthesis of thiophene derivatives catalysed by eggshell/Fe_3_O_4_.

Zargari [132] et al. (2024) reported a green and eco-friendly eggshell/Fe_3_O_4_ biocatalyst, prepared by impregnating waste eggshells with Fe_3_O_4_ nanoparticles. The eggshell/Fe_3_O_4_ catalyst was applied to the MCRs of different aromatic aldehydes, elemental sulphur (S_8_), and α-cyano ester for the synthesis of 2-aminothiophene derivatives. Reactions were carried out under microwave irradiation in ethanol to obtain the desired products in 82–97% yields within 10 min, as illustrated in Scheme 40. In addition, the biocatalyst eggshell/Fe_3_O_4_ exhibited excellent catalytic performance and recyclability, showing no significant loss in efficiency over five successive runs.

### 5.11. Microwave-Assisted Synthesis of Benzoxazine Derivatives

Benzoxazines are important heterocyclic scaffolds in organic chemistry and are widely present in approved pharmaceuticals. Benzoxazine motifs are associated with diverse biological activities, including antiallergic, antimycobacterial, antimicrobial, and antifungal properties [133,134].

**Scheme 41:**
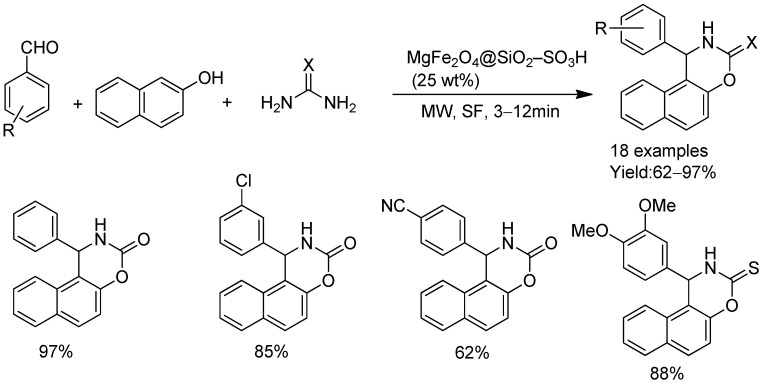
Preparation of benzthioxazinone and benzoxazinone derivatives catalysed by MgFe_2_O_4_@SiO_2_–SO_3_H- MNPs.

Salunkhe [135] et al. (2018) reported the production of SO_3_H–functionalized silica-coated MNPs (MgFe_2_O_4_@SiO_2_–SO_3_H) as an effective heterogeneous catalyst. The catalyst was successfully applied in the microwave-aided MCRs of bioactive benzthioxazinone and benzoxazinone derivatives from aldehydes, β-naphthol, and thiourea/urea under solvent-free conditions, affording products in 62–97% yields (Scheme 41). The catalyst was efficiently recycled for up to five successive runs with no appreciable decline in catalytic efficacy.

**Scheme 42:**
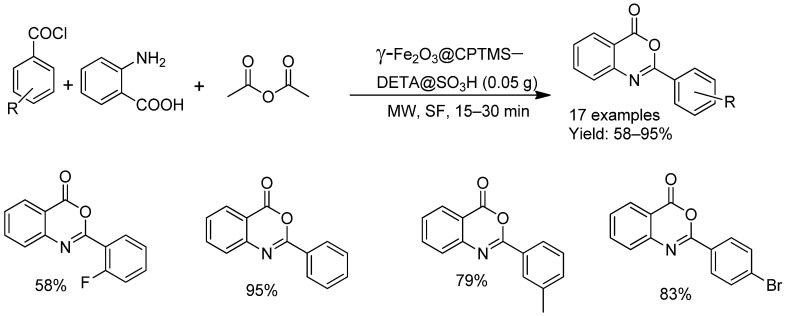
Preparation of benzoxazinone derivatives using γ–Fe_2_O_3_@CPTMS-DETA@SO_3_H.

Norouzi [136] et al. (2023) presented a new inorganic–organic superparamagnetic nanohybrid catalyst, γ-Fe_2_O_3_@CPTMS-DETA@SO_3_H, in which sulfonated sulfuric acid functionalities are immobilized on a magnetic support, establishing an environmentally friendly and efficient heterogeneous nanocatalyst. This catalyst was successfully used in the one-pot, 3CR of benzoxazinone derivatives via the condensation of various acyl chlorides, anthranilic acid, and acetic anhydride under solvent-free conditions the assistance of microwaves, yielding products in the range of 58–95% (Scheme 42). In addition, the catalyst was easily recoverable through magnetic decantation and was reusable for up to six cycles with no significant activity loss, demonstrating simplicity and sustainability.

### 5.12. Microwave-Assisted Synthesis of Benzodiazepine Derivatives

Benzodiazepines and their derivatives are bicyclic heterocycles consisting of a seven-membered diazepine ring fused to a benzene ring, containing two nitrogen atoms at distinct positions. This class of compounds has been widely used as antidepressant, hypnotic, analgesic, sedative, anticonvulsant, anxiolytic, and anti-inflammatory agents [137,138].

**Scheme 43:**
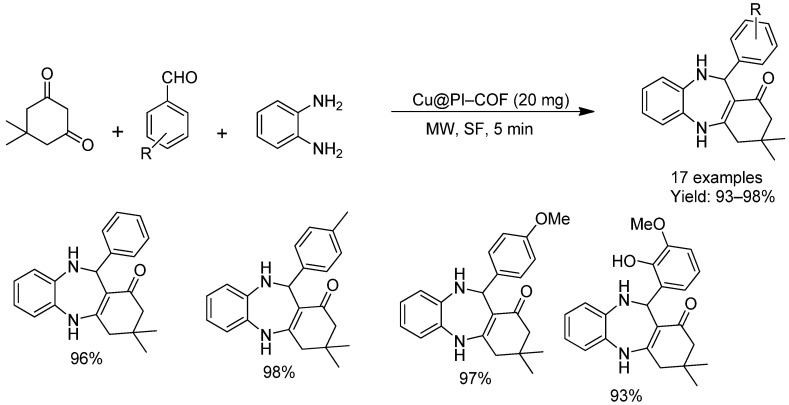
Synthesis of benzodiazepine derivatives catalysed by Cu@PI–COF.

Pourghasem [139] et al. (2023) reported copper (II)-anchored polyimide-linked covalent organic frameworks (Cu@PI-COF) as an efficient heterogeneous nanocatalyst. This catalyst was effectively applied in a one-pot MCR for the preparation of 1,5-benzodiazepines (Scheme 43) from *o*-phenylenediamine, aromatic aldehydes, and dimedone under microwave-assisted, solvent-free conditions, affording excellent yields (93–98%). The protocol offers several advantages, including high product purity, operational simplicity, easy work-up, short reaction times, high atom economy, and catalyst recyclability. Remarkably, the Cu@PI-COF catalyst could be reused for up to five repeated runs without substantial loss of catalytic action.

### 5.13. Microwave-Assisted Synthesis of Tetrazole Derivatives

Tetrazoles are synthetic heterocyclic compounds characterized by a five-membered ring containing one carbon and four nitrogen atoms. Tetrazoles and their derivatives display a wide range of biological actions, including antifungal, antiviral, antibacterial, antitubercular, hypoglycaemic, cyclooxygenase inhibitory, anti-inflammatory, antinociceptive, and anticancer properties [140,141,142].

**Scheme 44:**
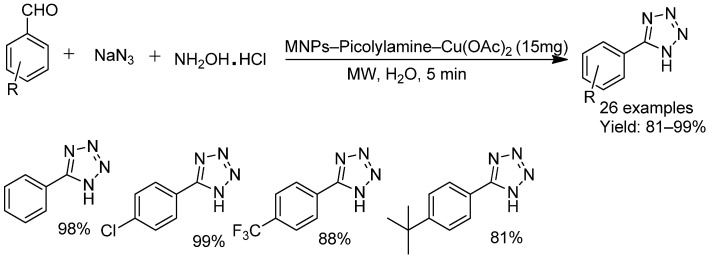
Preparation of tetrazole derivatives catalysed by MNPs–picolylamine–Cu(OAc)_2_.

Alexis Ramírez-Coronel [143] et al. (2024) reported the fabrication of a magnetic MNPs–picolylamine–Cu(OAc)_2_ nanocomposite via the immobilization of Cu(II) acetate on picolylamine-functionalized Fe_3_O_4_ NPs. The catalyst efficiently promoted a microwave-assisted one-pot 3CR preparation of 1*H*-tetrazole derivatives from aldehydes, sodium azide and hydroxylamine hydrochloride in water (Scheme 44). The Lewis acidic Cu(II) centres facilitated nitrile oxide formation and subsequent [3 + 2] cycloaddition, affording the required products in 81–99% yields. The magnetically recoverable catalyst was reclaimed for up to eight series without considerable loss of activity, highlighting its robustness and green credentials.

### 5.14. Microwave-Assisted Synthesis of Pyranopyrazole Derivatives

Pyranopyrazole moieties and their annulated systems constitute an important class of fused heterocycles that have attracted considerable interest due to their diverse biological and pharmacological activities. Reported activities include antibacterial, antioxidant, anti-inflammatory, antifungal, antiproliferative, antidepressant, anticancer, antimalarial, and anti-Alzheimer effects, as well as applications as biodegradable agrochemicals [144,145,146].

**Scheme 45:**
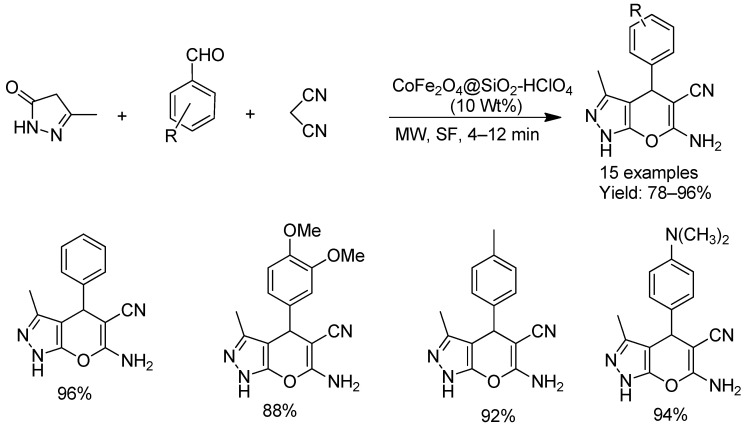
Synthesis of pyranopyrazoles using CoFe_2_O_4_@SiO_2_-HClO_4_ NPs.

Thakare [147] et al. (2023) described the preparation of a magnetically recoverable CoFe_2_O_4_@SiO_2_–HClO_4_ nanocatalyst showing excellent catalytic activity. A microwave-assisted synthesis of pyranopyrazole derivatives was successfully conducted using the said catalyst in a multicomponent reaction involving various aldehydes, 5-methyl-2,4-dihydro-3*H*-pyrazol-3-one, and malononitrile, yielding the products in 78–96% yields (Scheme 45). The regained catalyst could be efficiently reused for four repeated cycles under identical reaction conditions. In addition, the synthesized compounds demonstrated promising antimicrobial activity.

### 5.15. Microwave-Assisted Synthesis of Indole Derivatives

The indole ring system is one of the most widely occurring heterocycles in nature and serves as a key structural motif in numerous pharmaceutical compounds. Indole derivatives exhibit a broad range of biological activities, including anticonvulsant, antidepressant, antifungal, anti-inflammatory and antiviral properties [148]. Moreover, they play a significant role in the development of novel antitumour agents and HIV inhibitors [149].

**Scheme 46:**
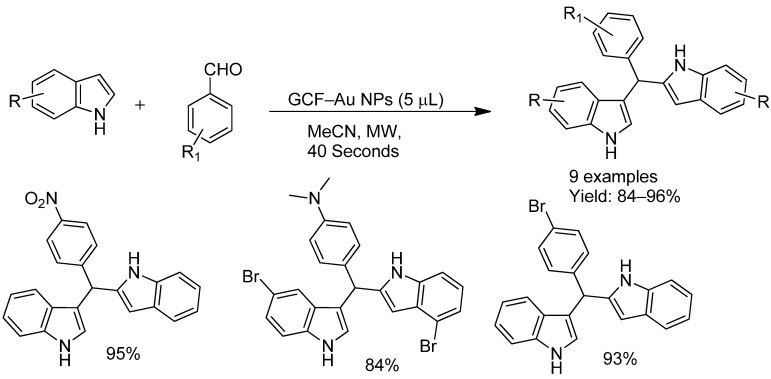
Synthesis of indole derivatives catalysed by Au NPs.

Gohain [150] et al. (2021) reported the green synthesis of gold nanoparticles (Au NPs) through an eco-friendly biogenic method using the aqueous fruit extract of *Garcinia cowa* at room temperature. The plant extract served as both a reducing and stabilizing agent, imparting excellent stability to the colloidal Au NPs. The synthesized nanoparticles were employed as a homogeneous catalyst for the coupling of substituted indoles with aromatic aldehydes to produce bisindolylmethanes (Scheme 46). The reaction was performed under microwave irradiation in acetonitrile, affording the desired products within 40 s in excellent yields of 84–96%. This strategy significantly improved the sustainability of the process by enabling rapid and efficient product formation.

### 5.16. Microwave-Assisted Synthesis of Benzoxazole Derivatives

Benzoxazole is a heterocyclic compound consisting of a bicyclic ring system in which an oxazole ring and a benzene ring are fused together. Derivatives of benzoxazole are significant heterocyclic frameworks found in many drug molecules and drug candidates. They display a variety of biological activities such as antifungal, antitubercular, anticancer, anti-inflammatory, analgesic, antitumour, and antibacterial activities, which have sparked considerable research in the discovery of novel drug candidates [151,152].

**Scheme 47:**
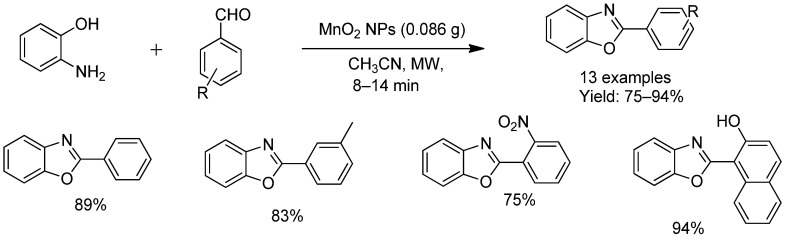
Synthesis of benzoxazole derivatives catalysed by MnO_2_ NPs.

An efficient and facile one-pot approach for the synthesis of 2-arylbenzoxazoles by coupling o-aminophenol with aromatic aldehydes using microwave irradiation was developed by Hossein Naeimi [153] et al. in 2017. The reaction was performed in the presence of MnO_2_ nanoparticles as oxidizing agents to afford 2-arylbenzoxazoles in good to excellent yields of 75 to 94% (Scheme 47).

### 5.17. Microwave-Assisted Synthesis of Triazole Derivatives

Triazoles are significant heterocyclic compounds that are utilized in medicine, agrochemicals, and material science. Moreover, the triazole ring is a significant structural component of several drugs that display a variety of pharmacological activities, such as anti-inflammatory, antifungal, antiviral, antidepressant, anticancer, and anti-allergic properties [154,155].

**Scheme 48:**
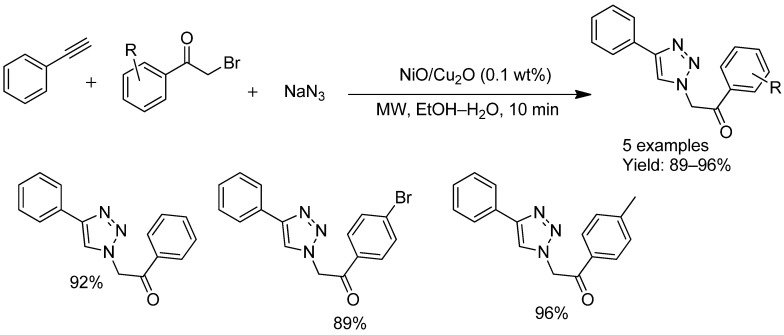
Synthesis of triazole derivatives catalysed by NiO/Cu_2_O NCs.

Attia [156] et al. (2024) reported the synthesis of Cu_2_O nanoparticles (Cu_2_O NPs) and NiO/Cu_2_O nanocomposites (NCs) and evaluated their catalytic activity in click reactions for the synthesis of 1,4-disubstituted 1,2,3-triazole derivatives (Scheme 48). The NiO/Cu_2_O NCs exhibited superior catalytic performance compared to Cu_2_O NPs. The reaction of benzoyl bromides, phenylacetylene, and NaN_3_ in the presence of sodium ascorbate in an ethanol–water medium under microwave irradiation afforded the desired triazole products in excellent yields (89–96%). Furthermore, the catalyst showed good recyclability and was reused for five consecutive cycles without significant loss of activity.

Nanoparticles catalysed multicomponent reactions carried out under conventional heating conditions involve slow and non-uniform heating, thus requiring longer reaction times and higher energy consumption. On the other hand, microwave irradiation allows for fast and uniform volume heating, as well as strong interactions between nanoparticles and reactants, resulting in faster reaction rates, higher yields, and better selectivity, as shown in Table 2.

## 6. Mechanistic Actions in MW–Nanoparticle-Catalysed MCRs

The mechanistic interaction of NPs under microwave irradiation is depicted in Table 3. The adsorption of the carbonyl substrate begins with the binding of the carbonyl-containing reactant (aldehyde or ketone) to the nanoparticle surface through coordination to Lewis acidic metal sites or hydrogen bonding to surface functional groups, thus increasing the local concentration of the reactant at catalytic sites. Subsequent interaction with surface metal or metal oxide sites leads to polarization of the C=O bond, thus increasing the electrophilicity of the carbonyl carbon. Under microwave irradiation, localized interfacial heating further enhances this activation process by increasing molecular mobility and surface reaction rates.

**Figure 1:**
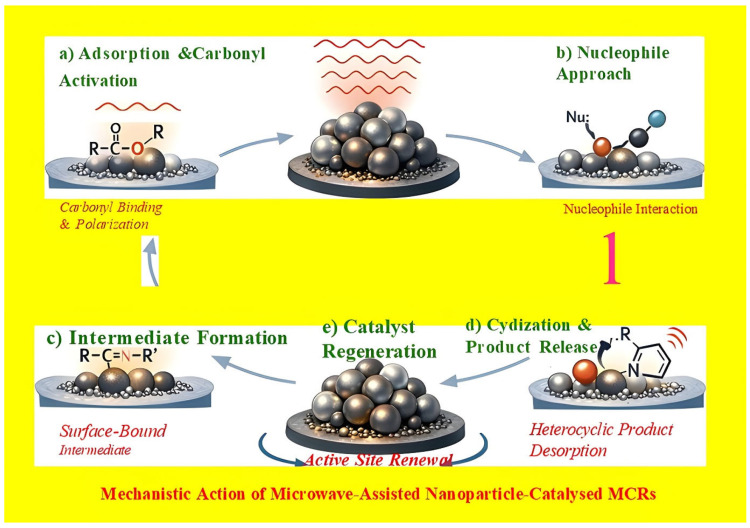
Mechanistic actions in MW-assisted MCRs catalysed by NPs.

The nucleophilic partner, such as an amine, an enolizable β-dicarbonyl compound, or an active methylene species, approaches the activated carbonyl while simultaneously interacting with the catalyst surface. Under microwave irradiation, rapid heating enhances effective collisions and proper orientation of the reactants. This results in the formation of a key surface-bound intermediate, such as an iminium ion, Knoevenagel adduct, or enamine, with the nanoparticle surface stabilizing charged or polar transition states and thus reducing the activation energy barrier for subsequent bond-forming steps. Finally, intramolecular condensation and cyclization reactions occur on the catalyst surface, followed by desorption of the heterocyclic product, thus regenerating the active catalytic sites for the next reaction cycle, as graphically represented in Figure 1.

## 7. Green Metrics and Sustainability Analysis of MCRs Catalysed by NPs Under MW Irradiation

Microwave-assisted multicomponent reactions mediated by nanoparticles are considered green reactions, but sustainability requires more data than just yield and reaction speed. The assessment of chemical transformations using green chemistry metrics is now considered to be of prime importance in evaluating the environmental impact of synthetic transformations. Atom economy (AE), E-factor, reaction mass efficiency (RME), and process mass intensity are some of the commonly used metrics for evaluating the efficiency of chemical transformations and their environmental impact. Atom economy is defined as the fraction of atoms from all reactants incorporated into the desired product, whereas the E-factor is defined as the mass of waste produced per mass of desired product. On the other hand, reaction mass efficiency is a function of yield and atom economy.

Considering the case of nanoparticle-catalysed and microwave-assisted multicomponent reactions, these metrics demonstrate the superiority of these reactions in comparison to conventional reactions. Multicomponent reactions are known to have high atom economy since all the reactants are incorporated into a single product, whereas microwave-assisted reactions are known to reduce reaction times and thus increase reaction efficiency. The use of nanoparticle catalysts is also beneficial in the context of sustainability because they show high catalytic activity and selectivity, as well as good recovery and recyclability. All the above characteristics are very consistent with the principles of green chemistry. Green chemistry metrics for MW-assisted nanocatalysed MCRs are given in Table 4 and Table 5.

The development of environmentally benign synthetic methodologies requires a quantitative evaluation of the sustainability of chemical processes. While catalyst recyclability and reduced reaction time are often considered indicators of green chemistry, a more rigorous assessment requires the use of green chemistry metrics that evaluate the overall material efficiency and environmental impact of a reaction. Among the most widely used metrics are the environmental factor (E-factor), reaction mass efficiency (RME), and process mass intensity (PMI). These parameters provide valuable insight into the efficiency of chemical transformations by accounting for the mass of reactants, catalysts, solvents, and other auxiliary materials involved in the process.

The environmental factor (E-factor) is defined as the ratio of the mass of waste generated to the mass of the desired product obtained. This metric was introduced to evaluate the environmental impact of chemical manufacturing processes, particularly in the pharmaceutical and fine chemical industries. A lower E-factor value indicates a more environmentally favourable process because less waste is produced per unit of product.

*E-factor* = *the ratio of the total mass of waste generated in a chemical reaction to the mass of the final product*

Waste in this context includes all materials that do not form part of the final product, such as solvents, excess reagents, and by-products. In many organic reactions, solvents contribute significantly to the total waste generated and therefore strongly influence the overall E-factor of the process. Another important parameter is the reaction mass efficiency (RME), which evaluates the fraction of reactant mass that is incorporated into the desired product. Unlike atom economy, which considers only the stoichiometric equation, RME incorporates the experimental yield and therefore provides a more realistic measure of reaction efficiency.

*RME* = *mass of desired product/total mass of reactants* × 100

Higher RME values indicate a more efficient utilization of starting materials, which leads to reduced waste generation and improved sustainability of the reaction.

The process mass intensity (PMI) is another widely used metric that evaluates the total mass of materials used in a process relative to the mass of the final product. PMI considers all materials involved in the reaction, including solvents, reagents, catalysts, and work-up materials. Consequently, it provides a comprehensive assessment of material efficiency.

*PMI* = *total mass of all materials used in the process/mass of product*

In most synthetic protocols, solvents represent the largest mass contribution to PMI. Therefore, the use of solvent-free conditions or environmentally benign solvents such as water or ethanol can significantly reduce PMI values and improve the overall sustainability of a reaction.

Microwave-assisted nanoparticle-catalysed multicomponent reactions reported in the literature frequently demonstrate several characteristics that can contribute to improved green metrics. These include high product yields, reduced reaction times, lower catalyst loading, and minimized solvent usage. Microwave irradiation enables rapid and uniform heating of the reaction medium, which often leads to shorter reaction times compared with conventional thermal heating. This reduction in reaction time may also decrease the overall energy intensity of the process. Furthermore, multicomponent reactions (MCRs) inherently exhibit high atom economy because multiple bonds are formed in a single synthetic step, minimizing the number of purification stages and reducing the generation of intermediate waste streams. When combined with recyclable nanoparticle catalysts and microwave irradiation, these reactions may offer significant advantages in terms of material efficiency and energy consumption.

Nevertheless, it should be noted that a complete quantitative evaluation of green metrics is not always possible for every literature report due to the limited availability of detailed mass balance data. However, the available information clearly suggests that microwave-assisted nanoparticle-catalysed multicomponent reactions often demonstrate favourable sustainability characteristics, particularly when solvent-free conditions, benign solvents, and recyclable catalysts are employed. Overall, the application of green chemistry metrics such as E-factor, reaction mass efficiency (RME), and process mass intensity (PMI) provides a more comprehensive framework for evaluating the environmental performance of synthetic methodologies. These metrics enable more reliable comparisons between conventional and emerging catalytic processes, and their comparative values are presented in Table 6.

## 8. Challenges and Future Perspectives

Microwave-assisted multicomponent reactions catalysed by nanoparticles have several advantages, but there are some drawbacks that need to be overcome for the sustainability of the process. The green nature of nanocatalysts has been overemphasized, as the synthesis of nanocatalysts can be energy-intensive and can involve toxic chemicals. The issues of stability, metal leaching, aggregation under microwave irradiation, and recyclability are still some of the major concerns. Moreover, the scalability of the microwave process is difficult due to the low penetration depth, inhomogeneous heating of bulk samples, and high cost of commercial microwave reactors. The lack of clarity about the mechanism of microwave-surface interactions is still a concern for the design of nanocatalysts, and the absence of a standardized green parameter makes it difficult to compare the sustainability of the process.

The future of NP-MW-assisted MCRs should be focused on earth-abundant and biogenic nanomaterials, low-energy or in situ nanoparticle synthesis, and the design of continuous-flow microwave reactors. More attention should be paid to the mechanistic understanding of microwave-surface interactions, and advanced characterization and computational resources will be helpful in this aspect. The use of quantitative green parameters and life-cycle assessment is necessary to validate green claims. Recent advances in green synthesis using plant biomass waste, such as fruit husks, leaves and agricultural residues, have demonstrated their feasibility as sustainable nanocatalyst sources.

## 9. Conclusions

Nanoparticle-catalysed microwave-assisted multicomponent reactions represent an efficient platform for the rapid synthesis of diverse heterocycles. The combination of high-surface-area nanocatalysts with volumetric microwave heating enables faster reaction rates, improved selectivity, and reduced solvent and time requirements compared to many conventional methods. A wide range of metal, metal oxide, magnetic, and hybrid nanomaterials has demonstrated versatility across different MCR classes, with the added advantage of catalyst recovery and reuse.

Nevertheless, the sustainability of these systems requires critical consideration. The environmental impact of nanoparticle preparation, potential metal leaching, catalyst deactivation, and the limited scalability of microwave reactors remain important challenges. Furthermore, many reports lack quantitative green metrics and detailed post-reaction catalyst characterization, underscoring the need for more rigorous evaluation.

Mechanistically, the cooperative effects of localized microwave heating and nanoparticle surface activation provide a rational basis for rate enhancement, yet deeper studies are needed to distinguish thermal from surface-specific microwave effects. Future advances should focus on earth-abundant and bio-derived catalysts, energy-efficient nanoparticle synthesis, scalable microwave technologies, and standardized sustainability metrics. With these developments, microwave-driven nanoparticle MCRs hold strong potential for more sustainable heterocyclic synthesis.

## Figures and Tables

**Table 1:** Comparison between conventional catalysts and nanoparticle catalysts.

Parameter	Conventional Catalysts (Bulk/Homogeneous)	Nanoparticle Catalysts
Surface Area	Relatively low	Very high surface-to-volume ratio
Active Sites	Limited catalytic active sites	Large number of accessible active sites
Reaction Rate	Moderate to slow	Significantly faster
Reaction Conditions	Often requires higher temperature and longer reaction time	Mild conditions with shorter reaction time
Product Yield	Moderate yield	Higher yield
Catalyst Recovery	Difficult for homogeneous catalysts	Easy recovery and recyclability
Energy Efficiency	Lower	Higher due to efficient microwave absorption
Selectivity	Moderate	Often improved selectivity

**Table 2:** Comparison of nanocatalysed MCRs under conventional heating and microwave irradiation.

Reaction Type	Nanocatalyst	MW Time	Conventional Time	Yield (%)	Solvent	Catalyst Reuse (Cycles)	Atom Economy
Biginelli reaction	PGO	5–10 min	20 min	95–98	Solvent-free	4	High
Pyran	Co–Ni Oxide	10 min	1 h	84–96	Solvent-free	12	Moderate–High
Benzodiazepine	Cu@COF	10 min	5 h	93–98	Solvent-free	5	High
Propargylamine	CuNPs@ZnO–PTh	6 min	2 h	78–97	Ethylene glycol	4	Moderate
Pyrido-pyrimidine	SiNPs (rice husk)	7 min	3 h	90–98	Water	8–9	High
Tetrazole	MNPs-Picolylamine-Cu(OAc)_2_	15 min	2 h	81–99%	Water	8	High
dioxolo-xanthenone	ZnO-β Zeolite.	4 min	3 h	84–95%	EtOH	4	Moderate–High
Indazole	S-LCCO NPs	4 min	20 min	84–94%	Solvent-free	4	Moderate–High
Thiophene	eggshell/Fe_3_O_4_	4–15 min	180 min	82–97%	EtOH	5	Moderate–High

**Table 3:** Microwave interaction with nanoparticles.

Effect	Illustration	Caption
Localized superheating	Hot spots around NP surface	NP absorbs MW energy more efficiently than bulk medium
Enhanced dielectric loss	Polar reactants clustering near NP	Increased local temperature at catalytic sites
Surface charge polarization	+/− charges on NP surface	Facilitates adsorption of polar intermediates

**Table 4:** Green chemistry metrics for MW-assisted nanocatalysed MCRs.

Metrices	Measured Parameter	Green Significance
Atom Economy (AE)	Efficiency of atom incorporation into product	Higher AE: less theoretical waste; MCRs typically show high AE
E-Factor	Amount of waste generated per mass of product	Lower value indicates greener process; solvent-free MW reactions reduce E-factor
Reaction Mass Efficiency (RME)	Practical mass efficiency considering yield	Combines AE and yield; reflects real process efficiency
Process Mass Intensity (PMI)	Total material used per mass of product	Lower PMI means fewer resources consumed
Carbon Efficiency (CE)	Fraction of carbon ending in product	Useful for carbon-rich heterocycle synthesis
Energy Efficiency	Energy required per unit product	MW reduces time but must be measured, not assumed
Catalyst Turnover Number (TON)	Catalyst productivity	Higher TON: less catalyst waste
Catalyst Recyclability	Reuse capability	Indicates long-term sustainability of nanocatalyst

**Table 5:** Green chemistry metrics in microwave-assisted nanocatalysed multicomponent reactions.

Nanocatalyst	Heterocycle	Reaction Conditions	Reaction Time	Yield (%)	Recyclability (Cycles)	Distinct Green Chemistry Attributes
CoFe_2_O_4_@SiO_2_–SO_3_H	Pyridine derivatives	Solvent-free, MW irradiation	Short (minutes)	86–92	5	Solvent-free, magnetic recovery
Fe_3_O_4_@niacin	Cyanopyridines	Water, MW irradiation	Rapid	73–95	6	Aqueous medium, bio-functionalized catalyst
H_3_PW_12_O_40_@nano-ZnO	Pyrimido-phenazine derivatives	Water, MW irradiation	Short	84–92	5	Non-toxic solid acid, aqueous medium
NiFe_2_O_4_@MCM-41@IL/Pt	Imidazo-pyrimidines	MW irradiation, short time	15–25 min	89–96	5	Magnetic separation, rapid synthesis
Ni(II)-Zeolite Y	Dihydropyrimidinones (DHPMs)	MW irradiation	Rapid	62–81	4	Heterogeneous catalyst, reduced waste
Phosphate-Graphene Oxide (PGO)	Pyrimidinones	MW irradiation	Short	91–98	5	High atom economy, reusable nanocomposite
ZnO–Co_3_O_4_–CuO nanocomposite	Polyhydroquinolines	PEG, 400 W MW	Short	88–94	6	Green solvent (PEG), multimetal synergy
NiFe_2_O_4_@ZnMn_2_O_4_	Tetrahydropyrimidines & polyhydroquinolines	Solvent-free, MW	Rapid	86–97	4	Solvent-free, magnetic recoverability
Fe_3_O_4_@meglumine-SO_3_H	Dihydropyrimidinones	H_2_O/EtOH (1:1), MW	Short	90–98	4	Green solvent mixture, magnetic reuse
Cu@Ag core–shell NPs	Octahydroquinazolinones	MeOH, MW	Short	58–96	5	Core–shell recyclable catalyst
Fe_3_O_4_@SiO_2_@L-glutamine	Benzo-imidazo-pyrimidines	Water, MW	Rapid	87–96	5	Bio-functionalized, aqueous medium
Cu(II)/PL-COF	Triaryl imidazoles	Solvent-free, MW	Short	93–98	5	Solvent-free, recyclable COF
Cr_2_O_3_ NPs	Imidazoles	Water, MW	Rapid	89–98	6	Plant-mediated synthesis, aqueous system
Graphene Oxide	Imidazolidinones	MW irradiation	Short	65–89	7	Metal-free catalyst, high stability
Fe_3_O_4_-MMT-K10	Pyran/Chromene derivatives	Solvent-free, MW	Short	86–94	6	Solvent-free, magnetic nanocomposite
Co–Ni Mixed Oxide	Benzo-pyrans	MW irradiation	Rapid	84–96	12	Bio-derived preparation, high durability
Fe_3_O_4_@CS@Schiff base@Cu	Propargylamines	MW irradiation	Short	65–97	6	Magnetic support, high retention of activity
AgNPs@g-C_3_N_4_	Propargylamines	Ethanol, MW	Rapid	89–97	6	Recyclable, eco-friendly solvent
Fe_3_O_4_@MCM-48@IL/Pd	Benzo-furo-phenazines	Solvent-free, MW	Short	85–93	6	Magnetic, solvent-free, dual functionality
MgFe_2_O_4_@SiO_2_–SO_3_H	Quinazolinones	Solvent-free, MW	Rapid	78–95	5	Magnetic separation, reduced solvent use
Zr-MOF (Zr/VitB_3_)	Tetrahydroxanthenes	MW irradiation	Short	58–95	3	MOF-based, reusable framework
Zr/P co-doped TiO_2_	Azlactones	MW irradiation	Short	89–96	Not reported	Reduced reaction time, improved efficiency
Eggshell/Fe_3_O_4_	2-Aminothiophenes	Ethanol, MW	10 min	82–97	5	Waste-derived biocatalyst
Cu@PI-COF	Benzodiazepines	Solvent-free, MW	Rapid	93–98	5	High atom economy, solvent-free
MNPs–picolylamine–Cu(OAc)_2_	Tetrazoles	Water, MW	Short	81–99	8	Aqueous medium, magnetic reuse
CoFe_2_O_4_@SiO_2_–HClO_4_	Pyranopyrazoles	MW irradiation	Rapid	78–96	4	Magnetically recoverable

**Table 6:** Comparison of microwave-assisted nanocatalytic reactions with conventional heating methods based on green chemistry metrics.

Entry	Reaction Type	Reaction Conditions	Catalyst System	Reaction Time	Yield (%)	Solvent System	Estimated E-Factor	PMI	Sustainability Observation
1	Multicomponent heterocycle synthesis	Conventional heating	No catalyst	8–12 h	35–50	Organic solvent	40–60	65–85	Low conversion and high waste generation due to prolonged heating
2	Multicomponent heterocycle synthesis	Microwave irradiation	No catalyst	2–3 h	45–60	Organic solvent	30–50	50–70	Moderate yield but still significant solvent contribution
3	Multicomponent heterocycle synthesis	Microwave irradiation	Cu nanoparticle catalyst	10–20 min	85–95	Ethanol/solvent-free	6–14	12–22	Dramatic improvement in efficiency, reduced solvent use and waste
4	A3 coupling reaction	Conventional heating	No catalyst	10–15 h	30–45	Organic solvent	45–70	70–95	Poor atom utilization and high environmental footprint
5	A3 coupling reaction	Microwave irradiation	Metal nanoparticle catalyst	15–30 min	80–92	Water/ethanol	10–18	18–28	Improved atom economy and reduced mass intensity
6	One-pot multicomponent synthesis	Conventional heating	No catalyst	6–10 h	40–55	Organic solvent	35–60	60–80	Higher waste formation and energy consumption
7	One-pot multicomponent synthesis	Microwave irradiation	Recyclable nanoparticle catalyst	10–25 min	88–96	Solvent-free/green solvent	6–12	10–20	Excellent sustainability performance with minimal waste

## Data Availability

Data sharing is not applicable to this article as no data were created or analysed in this study.
